# Metabolic Engineering of the Phenylpropanoid Pathway Enhances the Antioxidant Capacity of *Saussurea involucrata*

**DOI:** 10.1371/journal.pone.0070665

**Published:** 2013-08-14

**Authors:** Jian Qiu, Fenghua Gao, Guoan Shen, Chonghui Li, Xiaoyan Han, Qiao Zhao, Dexiu Zhao, Xuejun Hua, Yongzhen Pang

**Affiliations:** 1 The Key Laboratory of Plant Resources/Beijing Botanical Garden, Institute of Botany, the Chinese Academy of Sciences, Beijing, China; 2 The Key Laboratory of Biology and Genetic Resources of Rubber Tree, Rubber Research Institute, the Chinese Academy of Tropical Agricultural Sciences, Danzhou, Hainan, China; 3 Plant Biology Division, the Samuel Roberts Noble Foundation, Ardmore, Oklahoma, United States of America; Cankiri Karatekin University, Turkey

## Abstract

The rare wild species of snow lotus *Saussurea involucrata* is a commonly used medicinal herb with great pharmacological value for human health, resulting from its uniquely high level of phenylpropanoid compound production. To gain information on the phenylpropanid biosynthetic pathway genes in this critically important medicinal plant, global transcriptome sequencing was performed. It revealed that the phenylpropanoid pathway genes were well represented in *S. involucrata*. In addition, we introduced two key phenylpropanoid pathway inducing transcription factors (*PAP1* and *Lc*) into this medicinal plant. Transgenic *S. involucrata* co-expressing *PAP1* and *Lc* exhibited purple pigments due to a massive accumulation of anthocyanins. The over-expression of *PAP1* and *Lc* largely activated most of the phenylpropanoid pathway genes, and increased accumulation of several phenylpropanoid compounds significantly, including chlorogenic acid, syringin, cyanrine and rutin. Both ABTS (2,2′-azinobis-3-ethylbenzotiazo-line-6-sulfonic acid) and FRAP (ferric reducing anti-oxidant power) assays revealed that the antioxidant capacity of transgenic *S. involucrata* lines was greatly enhanced over controls. In addition to providing a deeper understanding of the molecular basis of phenylpropanoid metabolism, our results potentially enable an alternation of bioactive compound production in *S. involucrata* through metabolic engineering.

## Introduction

Snow lotus is a well-known and rare traditional Chinese medicinal herb, the aerial part of which has been used commonly to treat a wide spectrum of clinical diseases, including arthritis, stomachache, and gynecological diseases [1], [2]. The main bioactive compounds found in snow lotus are polyphenols, including phenolic acids, flavonoids and lignans. The potent antioxidant effects of these compounds are responsible for anti-inflammatory, antimutagenic and anti-tumor activities [1]–[3]. Our previous studies have shown that total polyphenol/flavonoid content correlates with assayed antioxidant activity in a variety of wild snow lotus species [4]. Among the few available endangered and rare wild snow lotus species in China, *S. involucrata* is of particularly high quality for medicinal purposes due to its relatively higher total polyphenol/flavonoid content and antioxidant activity [4].

Polyphenol/flavonoid compounds are important plant secondary metabolites for both plant defense and human health. They are derived from the general phenylpropanoid biosynthetic pathway, which is comprised of several biosynthetic branches, including lignin, stilbenes, flavonoids, and anthocyanins [5]. The biosynthesis of polyphenol/flavonoid has been extensively studied in the model plants *Arabidopsis*, petunia and maize [6], [7]. A ubiquitous regulation complex comprising MYB, bHLH and WD40, is structurally and functionally conserved among species for flavonoid biosynthesis [8], [9]. The *PAP1* (Production of Anthocyanin Pigment 1) gene encoding a MYB transcription factor from *Arabidopsis* is a key player involved in the anthocyanin biosynthetic pathway [10]. Over-expression of *PAP1* can effectively induce anthocyanin production in tobacco, hops, rose, *Salvia miltiorrhiza* and canola by regulating the related pathway genes, chalcone synthase (CHS), anthocyanidin synthase (ANS), flavonol synthase (FLS) etc [11]–[16]. The *Lc* (Leaf color) gene encodes a bHLH-type transcription factor identified from maize, which plays an important role in regulating anthocyanin production [17]. The ectopic expression of *Lc* can enhance anthocyanin and other flavonoid accumulation in *Lycopersicon esculentum* [18], petunia [19], *Medicago sativa* [20], *Caladium bicolor* [21] and *Malus domestica* [22]. In addition to expression studies of the individual genes, *PAP1* and *Lc* were also expressed simultaneously in an effort to increase anthocyandin production in *Arabidopsis* [23]. Given that PAP1 and Lc have been shown to successfully induce phenylpropanoid biosynthesis, are of two distinct gene families, and are derived from different species, these two transcription factors were here selected as high potential candidates for use as metabolic engineering tools in *S. involucrata*, which has not been modified genetically to generate whole transgenic plant previously.

In the present study, two key transcription factors (PAP1 and Lc) were selected to enhance phenylpropanoid production in *S. involucrata*. Expression of these transcription factors in transgenic *S. involucrata* greatly increased the levels of individual phenylpropanoids (including phenolic acids, lignins, flavonoids and anthocyanins), total phenolics, total flavonoids and antioxidant capacity. The transcriptome data of *S. involucrata* obtained in this study can push forward the efforts for the metabolic engineering of medicinal plants in terms of high-value products. Furthermore, our study demonstrates that *S. involucrata* would be an ideal system for studying natural product biosynthesis in medicinal plants.

## Results

### Transcriptome sequencing of wild *S. involucrata*

In order to obtain global transcriptome information for further investigation of the phenylpropanoid pathway in *S. involucrata*, Illumina sequencing was performed with pooled cDNA samples from leaf, stem and inflorescence organs (the medicinal parts) of wild *S. involucrata*. From 1.33 Gb of primary sequencing data, 56,151 unigenes with more than 200 bp in length (covering 28.3 Mb) were obtained after sequence cleaning and assembling with the SOAP *de novo* program (http://soap.genomics.org.cn). The raw transcriptome sequence information from the present study was deposited at the Sequence Read Archive (SRA) with accession No. SRA094934. Assembled unigenes were deposited at the NCBI Transcriptome Shotgun Assembly (TSA) database with accession No. JW881739 - JW918169.

**Figure 1:**
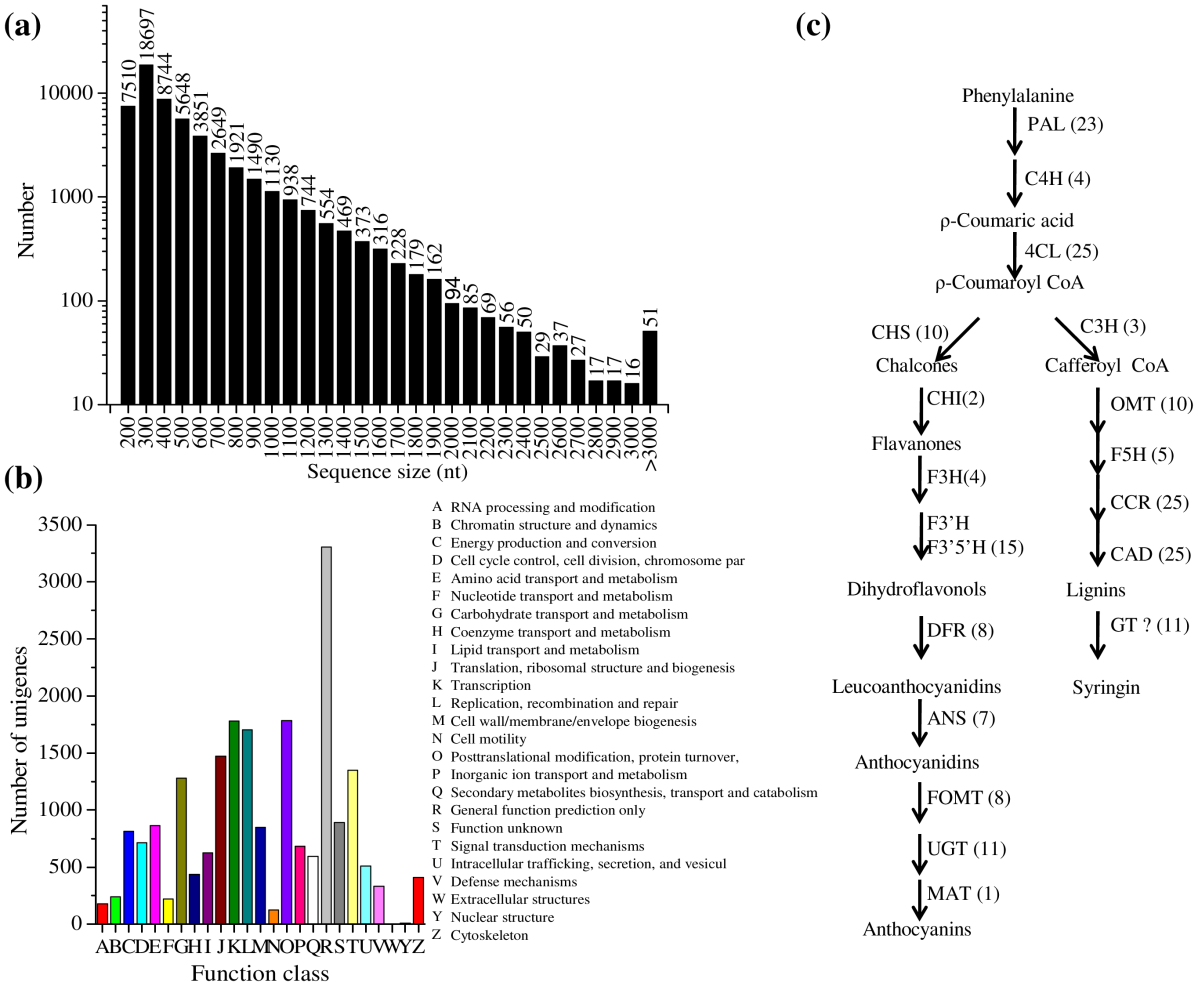
Sequence analysis of ESTs from *S. involucrata* by RNA-sequence. (a) Length distribution of Unigene in transcriptome of *S. involucrata*. (b) The unigene function annotation analyzed by the Cluster of Orthologous Groups. (c) Unigenes distribution in the flavonoid biosynthetic pathway. The abbreviations of the pathway enzymes are listed: PAL, Phenylalanine ammonia lyase; 4CL, 4-coumaroyl:CoA-ligase; C3H, coumarate 3-hydroxylase; CHS, chalcone synthase; CHI, chalcone isomerase; F3H, flavanone 3-hydroxylase; F3′H, flavonoid 3′-hydroxylases; F3′5′H, flavonoid 3′,5′-hydroxylases; DFR, dihydroflavonol reductase; ANS, anthocyanidin synthase; FOMT, flavonoid O-methyltransferase; UGT, UDP-glucuronosyl/glucosyl transferase; OMT, caffeic acid 3-O-methyl-transferase; F5H, Ferulate 5-hydroxylase; CCR, cinnamoyl-CoA reductase; CAD, cinnamyl alcohol dehydrogenase; GT, glucuronosyl glucosyl transferase; MAT, malony/acyltransferase.

The number of sequences decreased as the length of the unigenes increased, ranging from a large number of short (200 bp) unigenes to a small number of long (more than 3 kb) unigenes (Fig. 1a). The functional annotation of 21,166 unigenes was predicted and classed into 25 groups with the Cluster of Orthologous Groups (www.ncbi.nlm.nih.gov/COG) (Fig. 1b). In total, 592 unigenes were predicted to be involved in the biosynthesis, transport and catabolism of secondary metabolites (Fig. 1b).

In order to better understand the function of unigenes in *S. involucrata*, a blastX against the KEGG protein database was made on the assembled unigenes. A total of 18,361 unigenes were assigned to 125 pathways (Table S1). Our results showed that the largest five pathway groups were metabolic pathways (4109, 22.38%, ko01100), plant-pathogen interaction (1324, 7.21%, ko04626), spliceosome (1108, 3.03%, ko03040), biosynthesis of plant hormones (906, 4.93%, ko01070) and biosynthesis of phenylpropanoids (736, 4.01%, ko01060). Almost all of the previously characterized phenylpropanoid biosynthetic pathway genes were represented in *S. involucrata* (Fig. 1c, annotations of some unigenes were listed in Table S2), including genes encoding the early pathway enzymes PAL, C4H, 4CL, the lignin-specific pathway enzymes C3H, OMT, F5H, CCR and CAD, the anthocyanin-specific pathway enzymes DFR and ANS. In addition, several genes encoding flavonoid or lignin-modifying enzymes were found in the transcriptome data. These enzymes are most likely to be involved in the glucosylation, methylation and acylation of the phenylpropanoid compounds based on gene annotation.

### Optimization of embryogenic-callus induction

**Figure 2:**
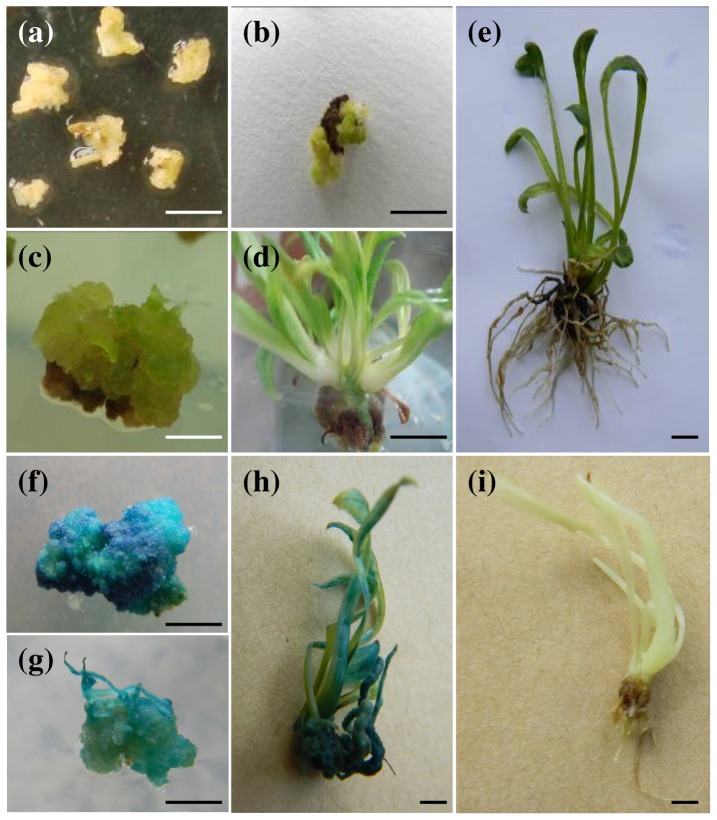
Genetic transformation and detection of *gus* transgene in *S. involucrata*. (a) calli induced from seeds. (b–e) Hygromycin-resistant calli, shoots (c–d) and roots (e) grown on selection medium. (f–h) GUS assay of corresponding transgenic calli (f), shoots (g) and plants (h) together with untransformed plants (i) ; Bars = 5 cm.

To facilitate functional genomics studies in *S. involucrata* using genetic transformation, seeds of *S. involucrata* with a relatively higher callus formation capability were used for embryogenic-callus induction. Seeds were grown on MS medium supplemented with various concentration of 2,4-D (2,4-dichlorophenoxyacetic acid, 1–5 mg l^−1^) and 6-BA (6-benzylaminopurine, 0 and 0.2 mg l^−1^). The yellow calli began to appear from the surface of seeds after 30 days of induction in darkness (Fig. 2a). The induction efficiency and callus growth rate were both correlated with the concentration of 2,4-D. When the concentration of 2,4-D was lower than 3 mg l^−1^, the induction rate was less than 20% (12.7% and 16.3% at 1 and 2 mg l^−1^, respectively, Table S3). When the concentration was increased to 3–5 mg l^−1^, the induction rate reached the highest value of 36% at 3 mg l^−1^, but dropped slightly to 34% and 33.7% at 4 and 5 mg l^−1^ concentrations, respectively. Cultures supplemented exclusively with 2,4-D exhibited slow growth. Calli growth could be increased marginally with 2,4-D concentrations higher than 3 mg l^−1^, but these cultures browned quickly. To deal with these problems, 6-BA at a concentration of 0.2 mg l^−1^ was supplied to stimulate the growth of calli (Table S3). The callus growth rate increased with increasing 6-BA concentrations up to 0.5 mg l^−1^, but calli treated with 6-BA above 0.2 mg l^−1^ lost their granular appearance, which is thought to indicate a loss of embryogenic potential. Additionally, it was found that the composition of the media affected callus induction. Calli grown on NB medium (N6 macronutrients+B5 micronutrients) showed a higher induction rate and better growth status than those grown on MS medium. Taken together, the optimal medium for callus induction of *S. involucrata* was the NB medium supplemented with 3 mg l^−1^ 2,4-D, while the optimal medium for subculture growth was the NB medium with addition of both 3 mg l^−1^ 2,4-D and 0.2 mg l^−1^ 6-BA (Table S3).

The sensitivity of *S. involucrata* callus to hygromycin was also tested, in preparation for subsequent efforts with genetic transformation (Fig. S1). It was found that a low concentration of hygromycin (less than 20 mg l^−1^) inhibited the growth of callus, but the callus remained alive. When the concentration of hygromycin was increased to more than 30 mg l^−1^, the callus eventually failed to survive at four weeks. As the concentration was increased to 50 mg l^−1^, the callus turned brown and died quickly within two weeks. Therefore, the 30 mg l^−1^ hygromycin concentration was chosen for the subsequent selection of transgenic *S. involucrata* plants.

### Generation of transgenic *S. involucrata* plants expressing *PAP1* and/or *Lc* genes

To test the feasibility of transformation, the callus of *S. involucrata* was transformed with *Agrobacterium* strain EHA105 harboring empty vector pCAMBIA1301. The calli were selected with hygromycin at a concentration of 30 mg l^−1^. The resistant callus, shoot and root grew well on the selection medium (Fig. 2b–f). PCR analysis with *gus*-gene specific primers confirmed the integration of *gus* gene into the genome of *S. involucrata* (Fig. S2). The activity of *gus* gene product was also detected by histochemical staining at different stages of transformation (Fig. 2g–i). These results suggest that the *Agrobacterium*-mediated genetic transformation system worked very well in *S. involucrata*.

**Figure 3:**
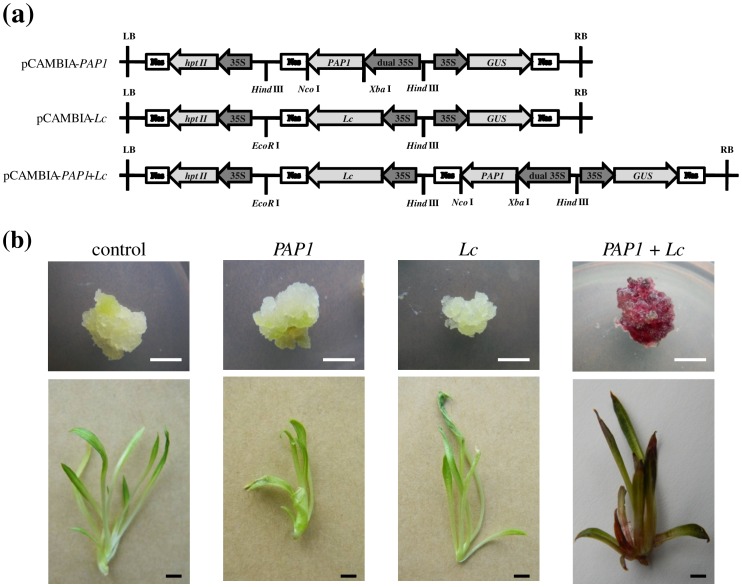
Schematic representation of T-DNA for binary vector plasmids used in this study and the phenotypes and gene expression levels of transgenic *S. involucrata*. (a) Schematic of T-DNA for vectors pCAMBIA-*PAP1* (upper), *Lc* (middle) and *PAP1*+*Lc* (bottom) used in transformation. LB, left border; RB, right border. (b) Phenotype of transgenic callus (upper panel) and plants (lower panel). The plasmids used for transformation were pCAMBIA1301, pCAMBIA-*PAP1*, pCAMBIA-*Lc*, pCAMBIA-*PAP1*+*Lc* from left to right.

Plasmids (pCAMIA-*PAP1*, pCAMIA-*Lc* and pCAMIA-*PAP1*+*Lc*) harboring the *PAP1* and *Lc* genes alone or in combination (schematic diagrams of T-DNA regions of transformation vectors were shown in Fig. 3a) were transformed into *S. involucrata* embryogenic calli via *Agrobacterium*-mediated genetic transformation. After two months selection with antibiotics, the calli transformed with pCAMBIA-*PAP1* or -*Lc* alone exhibited the same green color as the empty vector control lines (Fig. 3b, upper panel). Interestingly, only the transgenic lines transformed with pCAMBIA-*PAP1*+*Lc* exhibited purple pigmentation (Fig. 3b, upper right panel). All of the transgene-positive callus lines were transferred onto shoot regeneration medium. The shoots transformed with pCAMBIA-*PAP1* or -*Lc* alone exhibited the same green color as the empty vector control shoots (Fig. 3b, lower left panels). Interestingly, the shoots transformed with both *PAP1* and *Lc* exhibited a purple pigmentation phenotype throughout the entire shoot (Fig. 3b), indicating that *PAP1* and *Lc* together could induce anthocyanin production in *S. involucrata*.

### Expression of *PAP1* and *Lc* genes in transgenic *S. involucrata*

**Figure 4:**
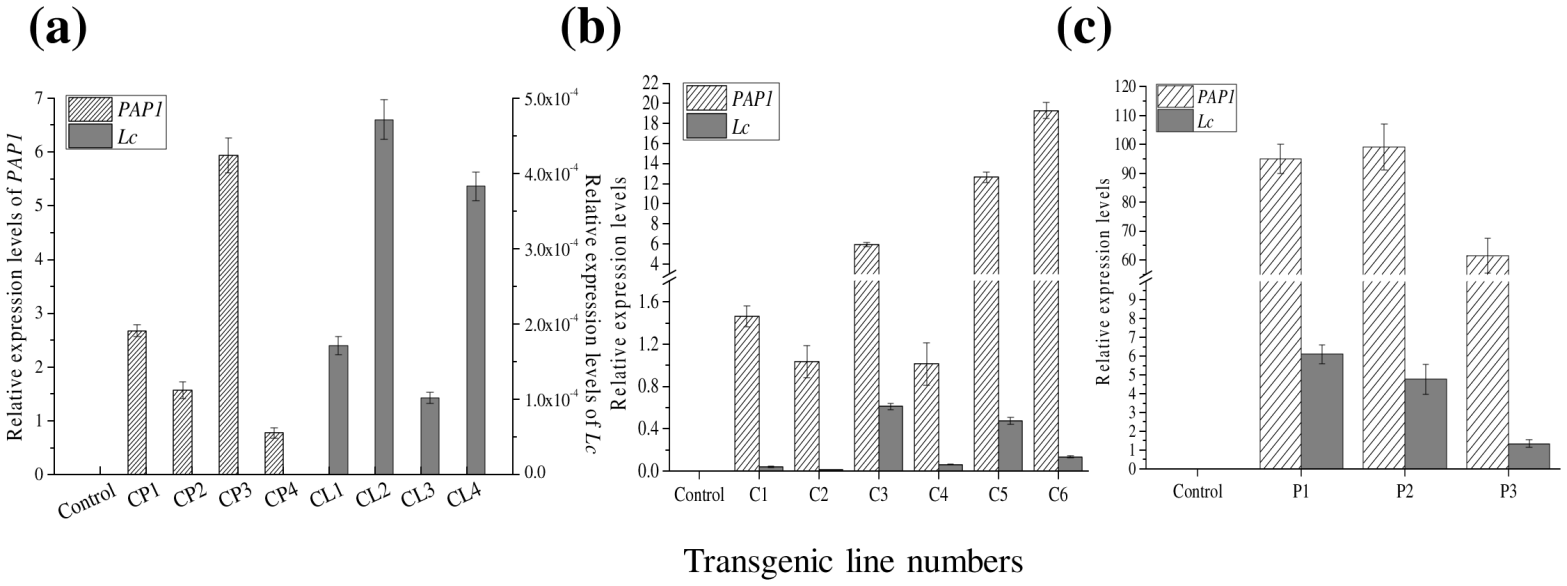
Relative expression levels of *PAP1* and *Lc* gene in transgenic plants. (a) Relative expression levels of *PAP1* or *Lc* in four transgenic *PAP1* callus lines (CP1–CP4) or *Lc* (CL1–CL4) alone. (b) Relative expression levels of *PAP1* and *Lc* in transgenic callus lines (C1–C6) co-expressing *PAP1* and *Lc*. (c) Relative expression levels of *PAP1* and *Lc* in *PAP1*+*Lc* transgenic plants. (Expression levels were relative to *GAPDH*).

The integration of *PAP1* and *Lc* genes into transformed calli and shoots were confirmed by PCR analysis with gene specific primers (Fig. S3). The expression level of these two genes was further measured by qRT-PCR using *GAPHD* (glyceraldehyde-3-phosphate dehydrogenase) gene as a housekeeping reference gene (Fig. 4). The expression of these two genes was undetectable in the control callus. The relative expression level of *PAP1* and *Lc* genes was between 1 to 6 (for *PAP1*), and below 0.0005 (for *Lc*), respectively (Fig. 4a). In the calli co-expressing *PAP1* and *Lc*, the expression levels of both *PAP1* and *Lc* were much higher than those in the lines transformed with a single gene (Fig. 4b). The relative expression level of *PAP1* was almost 20, while that of *Lc* gene was around 0.7 (Fig. 4c). The expression level of *Lc* in *PAP1*+*Lc* callus lines was 1000-fold higher than in the lines expressing only *Lc* (Fig. 4b). Interestingly, the expression level of *PAP1* and *Lc* reached nearly 100 and 6, respectively, in the shoots regenerated from the *PAP1*+*Lc* transformed calli. By comparison, the expression levels were 5- and 10-fold higher than those in the *PAP1*+*Lc* calli (Fig. 4c), indicating that these two genes were coordinately activated more effectively in shoots than in callus tissue.

### Effects of *PAP1* and *Lc* on phenylpropanoid pathway genes

**Figure 5:**
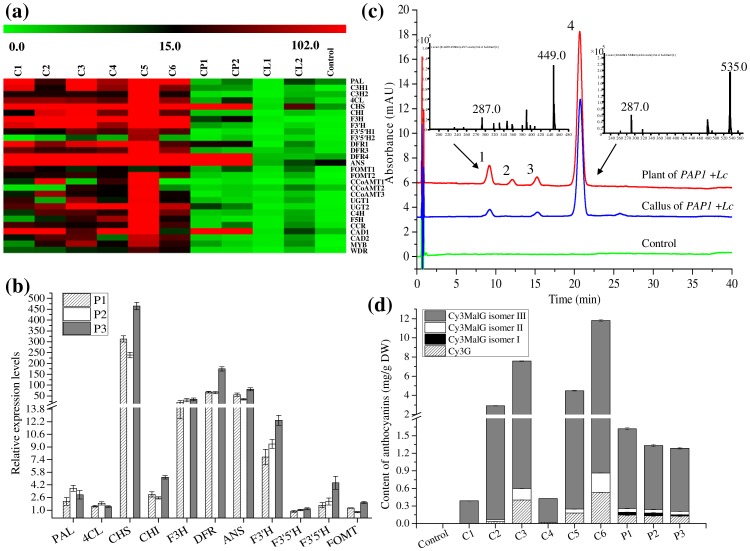
Detection of gene expression levels and analysis of anthocyanins in transgenic calli and shoots co-transformed with *PAP1* and *Lc*. (a) Transcript levels of 28 genes related to phenylpropanoid biosynthesis in six transgenic callus lines expressing *PAP1* and *Lc* (C1–C6), *PAP1* (CP1–CP2) or Lc (CL1–CL2) alone. (b) Expression levels of genes involved in flavonoid and anthocyanin biosynthetic pathways in transgenic plants (P1–P3). (c) Four anthocyanins detected at 515 nm by HPLC-UV-ESI-MS; 1, Cy3G; 2, Cy3MalGI; 3, Cy3MalGII; 4, Cy3MalGIII. Compounds were identified by their mass spectra (insert); (d) Anthocyanin content of control and transgenic calli and plants, C1–C6, transgenic calli, P1–P3, transgenic plants.

Using the available partial EST sequences of phenylpropanoid biosynthetic genes, qRT-PCR was performed to further examine the effects of *PAP1* and *Lc* on the entire phenylpropanoid/flavonoid pathway. The qRT-PCR results revealed that *CHS1*, *F3H*, *DFR4* and *ANS* were up-regulated coordinately in response to *PAP1* or *Lc* in the transgenic callus (Fig. 5a). When both *PAP1* and *Lc* were expressed, almost all of the pathway genes tested were up-regulated. In particular, all genes were increased more than 10-fold in line C5 (Fig. 5a). Among the 11 genes investigated in *PAP1* and *Lc* co-expressing plant lines, expression of *CHS1* gene was the most strongly induced (500-fold on average) in all three of the transgenic lines analyzed (Fig. 5b). In addition, *F3H*, *DFR4* and *ANS* showed 50-fold induction in the *PAP1*+*Lc* lines (Fig. 5b). *PAL*, *4CL1*, *CHI*, *F3′H*, which are all early pathway genes, were also clearly up-regulated in comparison to the non-transgenic control (Fig. 5b). The expression of *F3′5′H1,2* and *FOMT1* exhibited no significant change compared with controls (Fig. 5b).

### Anthocyanin levels in transgenic calli and shoots co-expressing *PAP1* and *Lc*

The presence of purple pigmentation was obvious in transgenic calli and shoots simultaneously expressing *PAP1* and *Lc* genes. This pigmentation was assumed to result from a high level of anthocyanin accumulation. In order to confirm this, HPLC and HPLC-MS were performed to detect and quantify the anthocyanin content. The HPLC-PDA chromatograms showed three peaks present in transgenic calli, and an additional fourth peak in transgenic shoots, but no obvious peaks were observed in non-transgenic controls (Fig. 5c). The four peaks were identified as anthocyanin derivatives, based on their UV spectra and mass spectrometry data (Fig. 5c). The four peaks were cyanidin glycosides, sharing the same cyanidin aglycone with *m*/*z* of 287. Peak 1 with molecular ion (M^+^) at *m/z* 449 was identified as cyanidin 3-*O*-glucoside (Cy3G, Fig. 5c left insert) by comparing with the authentic reference standard. The other three compounds were identified as isomers of cyanidin 3-malonylglucoside, and all of them shared a same molecular ion (M^+^) at *m*/*z* 535 (Fig. 5c right insert). They were designated as Cy3MalG I, II and III, respectively.

Compared with the non-transgenic control, the total anthocyanin content was dramatically increased by co-expressing *PAP1* and *Lc* genes (Fig. 5d). Cy3MalGIII (peak 4) was the major peak among the four anthocyanin compounds identified, and accumulated to a concentration ranging from 0.385 mg l^−1^ (line C1) to 10.958 mg l^−1^ (line C6) in independent transgenic calli. Cy3G and Cy3MalG I accumulation were relatively low (<0.5 mg l^−1^). In transgenic cluster shoots, the proportions of the three cyanidin 3-malonylglucoside isomers was similar to that in callus, but the total accumulation was much lower than those in the transgenic callus lines. Significant correlations were observed between the transcript level of *PAP1* and anthocyanin content (Cy3G, R = 0.814; Cy3MalGII, R = 0.821; Cy3MalGIII, R = 0.853).

### Additional phenylpropanoid compounds induced by *PAP1* and *Lc*

**Figure 6:**
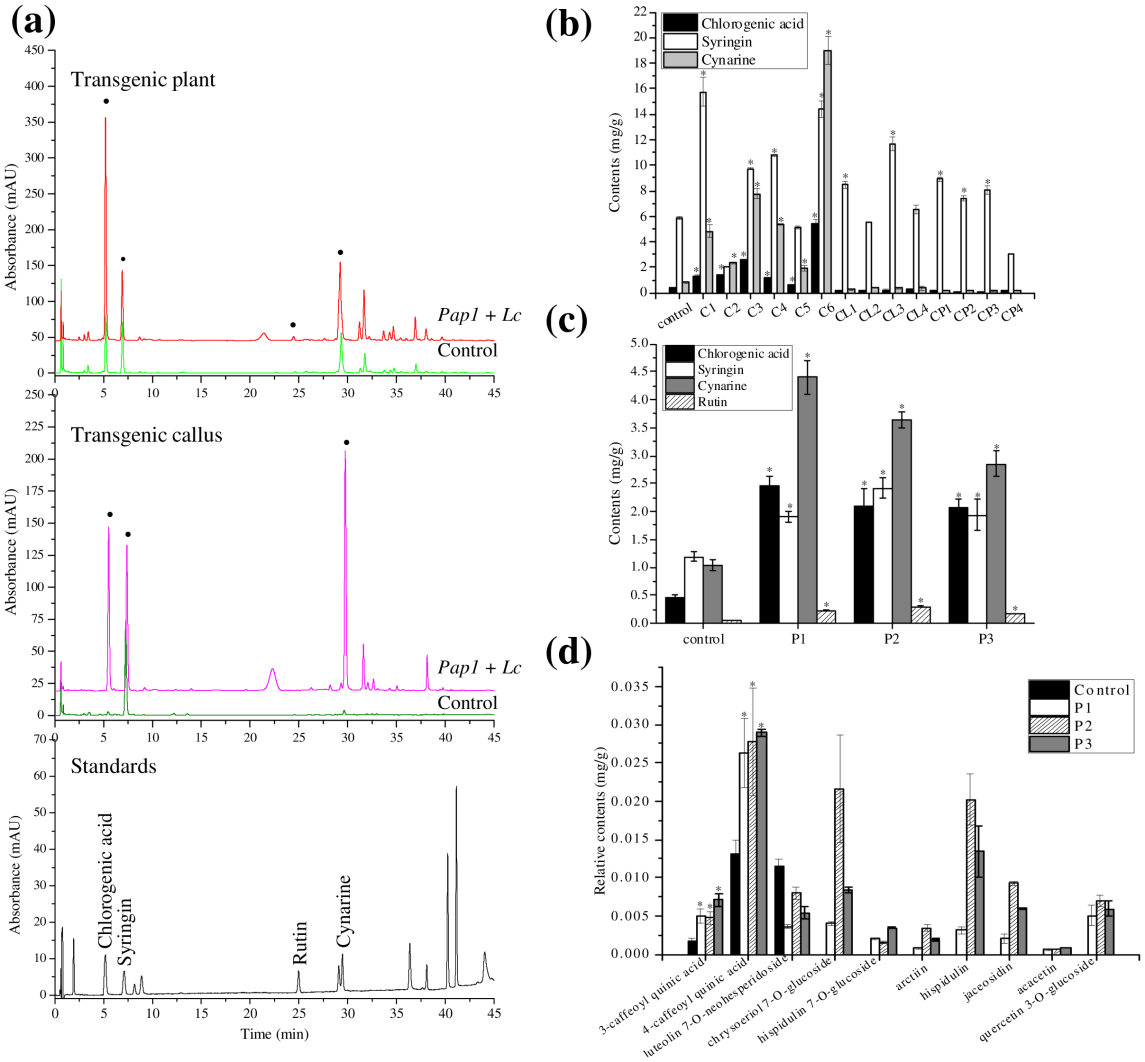
Detection of main phenylpropanoids in transgenic calli and plants of *S. involucrata* expressing *PAP1* and/or *Lc*. (a) HPLC-UV profile of phenylpropanoids from standards, control, transgenic calli and plants detected at 280 nm. (b) Content of chlorogenic acid, syringin, and cynarine in transgenic callus; Control, transformed with pCAMBIA 1301; C1–C6, callus lines co-expressing *PAP1* and Lc; CL1–CL4, callus lines expressing *Lc* alone; CP1–CP4, callus lines expressing *PAP1* alone. (c) Contents of chlorogenic acid, syringin, rutin and cynarine in transgenic plants (P1–P3) co-expressing *PAP1* and *Lc*. (d) Additional phenylpropanoids detected by LC-MS analysis in transgenic plants co-expressing *PAP1* and Lc. Values are expressed as peak area relative to a rutin standard curve. Asterisks indicate that a value is significantly different from that of the control by Student's t-test (P<0.05). The identification of the phenylpropanoid compounds are listed in Table S5.

The induction of several phenolic acid and flavonoid compounds were also observed in *PAP1*+*Lc* over-expressing calli and shoots, but not in control plants (Fig. 6a). The four major compounds induced in transgenic calli and shoots were: syringin, chlorogenic acid (5-caffeoyl quinic acid), cyanrine (1,5-caffeoyl quinic acid) and rutin (Fig. 6a). Syringin was the major monolignin component present in calli, the content of which varied among different transgenic lines,showing an increase of almost 2-fold in line C1 (Fig. 6b). The levels of two representative phenolic acids of chlorogenic acid and cynrine acid were greatly increased in transgenic calli compared with control calli (Fig. 6b). In particular, callus line C6 co-expressing *PAP1* and *Lc* accumulated the highest level of chlorogenic acid at 5.44±0.29 mg and cynrine acid at 18.97±1.1 mg per g fresh weight, and they were 15- and 24-fold higher compared with those of control calli (0.37 mg g^−1^ and 0.79 mg g^−1^, respectively). Line C6 also accumulated the highest anthocyanin levels with the strongest *PAP1* and *Lc* expression, indicating correlation of gene expression with the induction of metabolites. The content of syringin, chlorogenic acid and cynrine acid in calli transformed with *PAP1* or *Lc* alone showed no significant changes compared with control calli (Fig. 6b). For transgenic shoots, the contents of these three compounds were 2.21 mg g^−1^ (chlorogenic acid), 2.09 mg g^−1^ (syringin) and 3.64 mg g^−1^ (cynrine acid) on average, which were 1.7-, 4.8- and 3.5-fold higher than those of the control (Fig. 6c). Rutin was listed as a quality control standard for *S. involucrata* in the Chinese Pharmacopoeia, and its content in transgenic shoots was 5.0-fold higher than that of the control (Fig. 6c). Besides the above-mentioned major compounds, the contents of 3-caffeoyl quinic acid and 4-caffeoyl quinic acid in transgenic shoots were 3.5- and 2.1-fold higher than the control, respectively (Fig. 6d). In addition, seven other compounds were also detected in *PAP1*+*Lc* co-expressing shoots, including chrysoeriol 7-*O*-glucoside, hispidulin 7-*O*-glucoside, arctiin, hispidulin, jaceosidin, acacetin, and quercetin 3-*O*-glucoside. None of these compounds were detectable in the untransformed control shoots (Fig. 6d).

### Antioxidation activity analysis of transgenic *S. involucrata*

**Figure 7:**
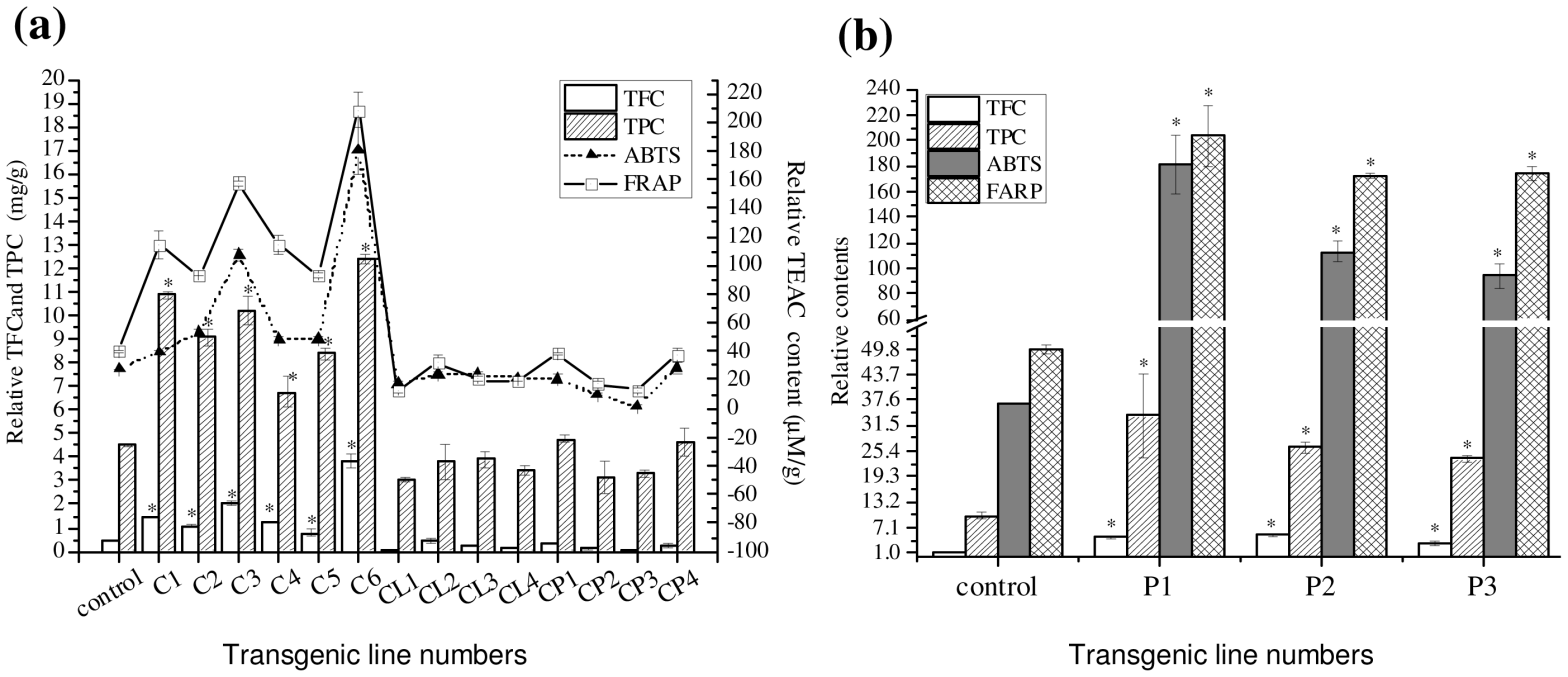
Total flavonoid and phenolic content, and the antioxidant activities of transgenic *S. involucrata* co-expressing *PAP1* and *Lc* genes. (a) callus lines expressing *PAP1* and/or *Lc* genes. CL1–CL4, callus lines expressing *Lc* alone; CP1–CP4, callus lines expressing *PAP1* alone, C1–C6, callus lines co-expressing *PAP1* and *Lc*. Control, callus transformed with vector pCAMBIA-1301. (b) plants co-expressing *PAP1* and *Lc* genes (P1–P3). Control, plant transformed with vector pCAMBIA-1301. Asterisks indicate that a value is significantly different from that of the control by Student's t-test (P<0.05).

To further determine the level of antioxidant activities in *PAP1+Lc* transgenic shoots, a variety of assays were conducted, including: total flavonoid content (TFC), total polyphenolic content (TPC) and trolox equivalent antioxidant capacity (TEAC). Our results revealed that the antioxidant capacity of *S. involucrata* in transgenic calli did not change when only *PAP1* or *Lc* was expressed alone (Fig. 7a), as indicated by nearly unchanged TFC and TPC levels. Whereas co-expression of *PAP1* and *Lc* together resulted in a significant enhancement of antioxidant capacity in *S. involucrata* calli and shoots, which demonstrated increased TFC and TPC levels (Fig. 7a–b). In particular, the TFC, TPC and TEAC levels in callus line C6 were 8-, 2.7-, 6.5- (ABTS assay) and 5.2- fold (FRAP assay) higher than those of the control (Fig. 7). In the transgenic shoots co-expressing *PAP1* and *Lc*, the average TFC, TPC and TEAC value of the three independent lines were 4.2-, 2.7-, 3.5- (ABTS assay) and 3.7-fold (FRAP assay) higher than those of the control, respectively (Fig. 7b). The antioxidant capacity was correlated positively with total anthocyanin content (ABTS, R = 0. 6949; FRAP, R = 0.6485), and accumulation of two individual compounds: chlorogenic acid (R = 0.9114; R = 0.8882) and cynrine acid (R = 0.7707; R = 0.7488).

## Discussion

In this study, metabolic engineering of the phenylpropanoid pathway by co-expression of the key transcription factors *PAP1* and *Lc* was successfully achieved in the snow lotus species *S. involucrata*. Previously, these two genes have been used for the genetic engineering of the phenylpropanoid pathway in other plant species, either alone or in combination [10]–[12], [14], [15], [22]–[24].

The *PAP1* gene from *Arabidopsis* encodes a MYB type transcription factor, which is a major component of the MYB-HLH-WD40 transcription factor complex that regulates the phenylpropanoid biosynthetic pathway. The MYB and bHLH type transcription factors have been widely used for the investigation and engineering of phenylpropanoid metabolism. When *PAP1* was over-expressed, most of the pathway genes were activated, resulting in the massive accumulation of purple pigments in all organs of *Arabidopsis* and tobacco [14], [25], or accumulation of these pigments in flowers and cones of hops [11]. In this study, *PAP1* expressed alone induced the expression of several pathway genes including *CHS1*, *DFR4*, *ANS* and *CAD1*, but did not significantly alter the phenylpropanoid content in *S. involucrata* (Fig. 5a–b). This might because *PAP1* failed to recognize its bHLH and/or WD40 transcription factor partner in *S. involucrata*. Similarly, when *PAP1* was over expressed in *Medicago sativa*, no anthocyanin accumulation was detected, while its ortholog *LAP1* from *Medicago truncatula* could activate the anthocyanin pathway in *M. sativa* [26]. Therefore, the failure of PAP1 to recognize its endogenous partners could explain its inability to induce anthocyanin biosynthesis in other plant species. It cannot be excluded that this may due to the low expression level of other unidentified genes that are essential in the pathway.

Similarly, the bHLH type transcription factor *Lc* expressed alone also failed to activate the biosynthesis of anthocyanins in *S. involucrata* (Fig. 3). However, previous studies have shown that the maize *Lc* exhibits a powerful transcriptional function in other plant species, such as *Arabidopsis thaliana* [23], *Malus domestica* [22], and *Caladium bicolor* [21]. Lc is believed to interact with, or induce intrinsic MYB-type transcription factors such as PAP1, the gene which is thought to cooperate with bHLH to control flavonoid biosynthesis [27]. Generally, bHLH proteins act as transcriptional activators or repressors, which facilitate MYB binding with the *cis* element of target genes. Therefore, bHLH proteins do not function alone in regulating a pathway, but require the involvement of a MYB transcription factor. The inability of maize *Lc* to regulate the flavonoid pathway in *S. involucrata* implies that Lc from maize cannot recognize the corresponding *S. involucrata* MYB transcription factor.

The phenylpropanoid pathway is a complex metabolic network, with many shared substrates and branches. A change in one branch can dramatically affect the other portions of the network. *Lc* over-expression in apple led to an increase of anthocyanin pigments in leaves and accumulation of proanthocyanidins, flavanols and many other phenylpropanoid compounds in a variety of organs and developmental stages [22]. Ectopic expression of *PAP1* in *Salvia miltiorrhiza* led to an increase in anthocyanin, total flavonoid and total phenolic content [15]. Ectopic expression of *PAP1* in rose increased anthocyanin as well as terpenoid scent compound content [16]. Our study revealed that the co-expression of *PAP1* and *Lc* in *S. involucrata* induced high levels of anthocyanin accumulation, as well as comparable levels of various other compounds including flavonoids, phenolic acid and lignin (Fig. 6).

There are several putative transcription factors (TF) in the *S. involucrata* transcriptome that are most likely related to phenylpropanoid biosynthesis. The Myb type TF MYB2 showed high homology (64% identity at amino acid level) to AtMYB90 [28], which regulates anthocyanin biosynthesis in *Arabidopsis*. MYB3 was homologous to MYB12 (66% identity) in *Arabidopsis*, which has been shown to up-regulate the expression of *CHS*, *F3H*, and *FLS* in *Arabidopsis* [29]. The bHLH1 from *S. involucrata* showed high similarity with *Transparent Testa 8* gene from *Arabidopsis*, which regulates the synthesis of flavonoids by interacting with MYB and WDR [30]. Thus, we inferred that MYB3 and bHLH1 control the synthesis and accumulation of flavonoids in *S. involucrata* through regulation of flavonoid biosynthetic genes. It is reasonable to assume that *S. involucrata* and previously studied plants share a similar regulatory mechanism and *cis* regulatory elements for control of phenylpropanoid biosynthesis, and our results strengthen this assumption.

The phenylpropanoid pathway generates an enormous array of metabolites through the modification of a small number of core intermediates. These intermediate compounds are modified by diverse enzyme classes such as glucosyltransferases, methyltransferases and acyltransferases [5], [31]. A large diversity of abundant flavonoid compounds, as well as examples of each of these modifying enzyme classes were all observed in *S. involucrata*. The flavonoid aglycones (cyanidin, quercetin, apigenin etc.) in *S. involucrata* were all glycosylated with various mono- and di-saccharides, including glucose, rhamnose, and rutinose [4]. These glycosylated flavonoids accumulated in *S. involucrata* at higher levels when PAP1+Lc were introduced (Fig. 5). Interestingly, eleven UDP-glucosyltransferases were discovered in the transcriptome according to their annotation (Fig. 1). Two of them, *UGT1* and *UGT2* were induced by PAP1+Lc in transgenic *S. involucrata* (Fig. 5a), implying they are the most likely enzymes responsible for the glycosylation of flavonoids in *S. involucrata*. Methylated cyanidins, in particular Cy3MalGIII was induced at the highest level by PAP1+Lc in *S. involucrata*, which presumably would contribute to the stabilization of cyanidin. Interestingly, an EST (unigene number 24950) was the only one annotated as MAT discovered from the transcriptome data, which is highly expressed in anthocyanin-accumulating inflorescence organs (unpublished data). This unigene is most likely the acyltransferase responsible for the addition of the malonyl moiety to glycosylated cyanidin in *S. involucrata*.

The quality of *S. involucrata* largely depends on its phenylpropanoid compound profiles, so we mainly focus on the phenylpropanoid biosynthetic pathway. By using KEGG pathway analysis, we were able to identify a range of putative genes involved in the phenylpropanoid biosynthesis pathway in *S. involucrata*. The number of unigenes assigned to the phenylpropanoid pathway was the fifth among the total number of unigenes, implying the phenylpropanoid biosynthesis is very active in *S. involucrata*. Almost all the phenylpropanoid pathway genes were represented in *S. involucrata* and many of these genes appeared to form multi-gene family (Fig. 1). Only one gene (*F3′H*) is missing in this pathway, which might be due to its low expression or insufficient sampling. With few of these pathway genes in *S. involucrata* have been characterized, the exact biosynthesis pathway and genes relevant to main phenylpropanoid compounds in *S. involucrata* still need to be confirmed by more genetic and biochemical evidences. Nevertheless, the transcriptome data sets obtained in this study via high through sequencing could facilitate functional genomics studies and in-depth analysis of secondary metabolite production in *S. involucrata*.

This study demonstrates the successful metabolic engineering of the phenylpropanoid pathway in *S. involucrata* through genetic modification with selected transcription factors. The highly induced phenylpropanoid compounds in transgenic *S. involucrata* are anthocyanins, chlorogenic acid and cyanrine, with their contents correlated with antioxidant activities (Fig. 6 and 7). Anthocyanins are well-documented potent antioxidant, which has been intensively investigated in various plant species [32], [33], [34]. The chlorogenic acid and cynarine in snow louts including *S. involucrata* showed good correations with their antioxidant activities [4]. The increased antioxidant activities of transgenic *S. involucrata* are primarily attributed by the introduction/increased anthocyanins, chlorogenic acid and cyanrine accumulation. Our engineering efforts therefore demonstrate that the antioxidant activity of *S. involucrata* was enhanced greatly as a result of the increase of health-promoting phenylpropanoid compounds. The establishment of the transgenic lines with high antioxidant activity represents a novel and potentially sustainable source of high quality *S. involucrata* for medicinal and commercial applications.

## Materials and Methods

### Plant materials and reagents

Seeds of *S. involucrata* were collected from a wild population in the Celestial Mountains at altitudes of 4000–5000 m (Hejing, Uighur of China), where no specific permission was required. We confirm that it is an open location to everyone and only seeds were collected, which did not involve endangered or protected species. All standards (3-caffeoylquinic acid, syringin, rutin, 1,5-dicaffeoylquinic acid, cyanidin-3-rutinoside, catechin and gallic acid etc.) used in this study were purchased from Sigma-Aldrich Co. (Shanghai, China). HPLC-grade methanol and acetonitrile were purchased from Thermo Fisher Scientific (Shanghai, China).

### Vector construction and transformation with *Agrobacterium tumefaciens*

The plasmid pSRLC349 containing the coding sequence of *Lc* gene (GenBank accession No. M26227), driven by CaMV 35S promoter, was kindly provided by Dr. Houhua Li (Institute of Biological Production Systems, Leibniz University of Hannover, Hannover, Germany). The *Lc* gene was subsequently subcloned into the binary vector pCAMBIA1301 by using compatible *Hind*III and *EcoR*I restriction enzyme sites to generate pCAMBIA-*Lc*.

The coding region of *Arabidopsis PAP1* (AT1G56650) was amplified from leaf cDNA with forward primers PAP1-F (located upstream of start codon ATG with an existing *Nco*I site) and the reverse primer PAP1-R with an introdued *Xba*I site. The PCR product was further ligated into the pRTL2 vector in the same sites (*Nco*I and *Xba*I digested) for cloning, followed by sequencing at Sangon Biological Engineering Technology & Services Co., Ltd. (Shanghai, China). The correct clone containing the *PAP1* gene driven by double CaMV 35S promoter from pRTL2 was excised with *Hind*III and inserted into both pCAMBIA1301 and pCAMBIA-Lc digested with the same enzyme, resulting in the binary vectors pCAMBIA-PAP1 and pCAMBIA-PAP1+Lc, respectively (Fig. 3a). These four vectors pCAMBIA1301, pCAMBIA-PAP1, pCAMBIA-Lc and pCAMBIA-PAP1+Lc all with the β-glucuronidase gene as reporter and the *hpt* II gene as selectable marker, were all introduced into *Agrobacterium tumefaciens* strain EHA105 through electroporation.

### Induction of callus, transformation and regeneration of *S. involucrata*

Surface-sterilized mature seeds of *S. involucrata* were maintained on MS or NB (N6 macronutrients and B5 micronutrients) medium supplemented with various concentrations of 2,4-D and/or 6-BA, and incubated at 25°C in darkness for callus induction. For transformation, following a two-month induction period, selected embryogenic calli were immersed in *Agrobacterium* suspension cultures harboring the four above-mentioned binary vectors under vacuum pressure for 5 minutes. After the removal of excess *Agrobacterium* with filter paper, the infected calli were transferred onto co-cultivation medium without antibiotics and incubated in darkness at 25°C for two and half days. These calli were then placed on selection medium (NB+3.0 mg l^−1^ 2, 4-D+0.5 mg l^−1^ 6-BA supplemented with 300 mg l^−1^ cefalexin and 30 mg l^−1^ hygromycin), and sub-cultured every two weeks. After two months, the resistant calli were maintained on regeneration medium (MS+1.0 mg l^−1^ 6-BA+0.1 mg l^−1^ NAA supplemented with 300 mg l^−1^ cefalexin and 30 mg l^−1^ hygromycin) under a 16 h light/8 h dark photoperiod with light intensity of 30–45 µmol m^−2^ s^−1^ at 25°C.

### Detection of transgenes by PCR and GUS assay

Genomic DNA was extracted from callus and plants by a modified CTAB method [35]. PCR amplification was performed with the primer pairs designed according to each individual gene (Primers are listed in Table S4). Amplified DNA fragments were separated by electrophoresis on a 1.5% agarose gel and stained with ethidium bromide. To confirmation T-DNA delivery, GUS activity was measured histochemically in staining solution (100 mM Na_2_HPO_4_-NaH_2_PO_4_ buffer pH 7, 1 mM K_4_Fe(CN)_6_, 0.5 mM EDTA, 0.1% X-glucuronide) at 37°C for 1–6 hrs and cleared in 75% ethanol.

### Transcriptome sequencing and quantitative Real-Time PCR

Total RNA from the aerial parts (leaves, stems and flowers) of the mature wild *S. involucrata* was isolated with TRIzol Reagent (Invitrogen, Carlsbad, CA, USA). After digestion with RNase-free DNase I (Takara, Dalian, China) for 30 min at 37°C to remove the residual DNA, the clean RNA was sent to BGI (Shenzhen, China) for cDNA library construction and Solexa sequencing (Illumina, San Diego, USA). The transcriptome was sequenced with the Illumina GA IIx platform according to the manufacturer's instructions (Illumina, San Diego, USA). *De novo* transcriptome assembly was carried out with the program-SOAPdenovo according to Li et al. [36], with default settings except K-mer value. Twenty eight-mer was chosen to construct the de *Bruijn* graph in the present study. Unigene sequences were searched against Nr and SwissProt protein databases using blastx (*E* value<10^−5^). Protein function information could be predicted from the annotations of the most similar protein from those databases. Unigenses were aligned to the COG database (http://www.ncbi.nlm.nih.gov/COG) to predict and classify possible functions with *E* value threshold of 10^−5^. Pathway assignments were carried out according to the Kyoto Encyclopedia of Genes and Genomes pathway database using BLASTx with *E* value threshold of 10^−5^.

Total RNA from the shoot of transgenic lines of *S. involucrata* for transcript profiling analysis was isolated with TRNzol reagent (TIANGEN, Beijing, China), followed by digestion with DNase I treatment (Takara, Dalian, China). First strand cDNA was synthesized from 2 µg total RNA using the PrimeScript® One Step RT-PCR Kit (Takara, Dalian, China). Quantitative Real-Time PCR (qRT-PCR) was performed on a Stratagene (Santa Clara, CA, USA) MP3000PCR platform using SYBR Green RT Master Mix (Takara, Dalian, China).

Primers used to amplify the phenylpropanoid pathway genes for qRT-PCR (Table S4) were designed using Beacon Designer 7 (PREMIER Biosoft) based on the EST sequences of *S. involucrata* transcriptome data. The ESTs were selected based on the highest sequence identity with their corresponding homologs from *Arabidopsis*. Triplicates of each reaction were performed, and *GAPDH* was chosen as an internal control for normalization after comparison with the expression of the reference gene *GAPDH*. Relative transcript profiles were calculated via the 2^−ΔΔCt^ method.

### Extraction, identification and measurement of phenylpropanoid compounds

For the extraction of phenylpropanoid compounds, the callus and plant material were freeze dried, ground into fine powder, and extracted with 70% aqueous methanol at 4°C for 24 hr, followed by centrifugation. The supernatants were then filtered through 0.2 µm Nylon filter membrane and stored at −40°C for further analysis. The extraction method was the same for anthocyanin extraction, except for the addition of HCl in the 70% methanol to a final concentration of 0.1%.

Analytical HPLC was carried out with the UltiMate™ 3000 RSLC rapid separation system from Dionex (Sunnyvale, CA, USA). The separation was performed with a ZORBAX RRHD Eclipse Plus C18 column (2.1×150 mm i.d. 1.8 µm, Agilent, Santa Clara, CA, USA) maintained at 30°C, at a flow rate of 0.4 mL·min^−1^. 10 µL of analyte was injected. The solvent system used was 0.2% aqueous formic acid (A) and acetonitrile (B) with a gradient of 0 min, 5% B; 20 min, 10% B; 35 min, 20% B; 40 min, 30% B; 50 min, 40% B; and 60 min, 5% B. UV–vis spectra were obtained by scanning from 190 to 800 nm. The chromatogram of each sample was recorded at 515 nm for anthocyanins and 280 nm for other phenylopropanoids.

HPLC-ESI-MS analysis was carried out on Waters Acquity UPLC/Xevo QTof (Waters Corporation, Milford, USA) equipped with an Acquity UPLC BEH C18 column (2.1×50 mm, i.d. 1.7 µm, Waters). The mobile phase were the same as mentioned above gradient elution program, 0–7 min, 5% B; 10 min, 15% B; 13 min, 35% B; 16 min, 50% B; 17 min, 80% B; and 18 min, 5% B; flow rage, 0.5 mL/min; temperature, 45°C; infection volume, 2 µL; MS detection: capillary voltage, 3.0 kV; cone voltage, 35 V; source temperature, 120°C; desolvation temperature, 400°C; cone gas flow, 50 L/h; desolvation gas flow, 800 L/h; collision energy, 15 V; detection mode, scan (*m/z* 100–1000; scan time 0.2 sec, interscan time 0.02 sec); both the positive and negative ion modes were employed. The data were recorded using Masslynx v4.1 software (Waters).

Metabolites were identified by the retention time and mass spectra data compared with authentic standards and their content was determined by linear regression with the corresponding standards. Anthocyanin and other polyphenol content were determined quantitatively with cyanidin-3-rutinoside and rutin as equivalents, respectively.

### Measurement of antioxidant activity

Total flavonoid content (TFC) and total phenolic content (TPC) were measured according to Qiu et al. [4] with the results expressed as milligrams of catechin and gallic acid, respectively. Antioxidant activity was determined by ABTS assay and FRAP assay, which has been previously described [4].

### Statistical analysis

Differences between means were located using Student's t-test by SPSS 17.0 (SPSS Inc., Chicago, IL, USA). Differences were considered statistically significant at p<0.05.

## Supporting Information

Figure S1 **Survival rate of** ***S. involucrata*** **calli on medium supplemented with different concentrations of Hygromycin.**
(DOC)

Figure S2 **PCR assay of selective marker** ***gus*** **gene in transgenic plants.**
(DOC)

Figure S3 **PCR assay of** ***PAP1*** **and** ***Lc*** **in transgenic plants.**
(DOC)

Table S1 **KEGG pathway assignment in** ***S. involucrata*** **.**
(DOC)

Table S2 **Functional annotations of some key unigenes involved in the phenylpropanoid biosynthesis.**
(DOC)

Table S3 **Growth and induction data of immature embryo calli.**
(DOC)

Table S4 **Primers used in this study.**
(DOC)

Table S5 **Identification of phenylpropanoid compounds in** ***S. involucrata*** **.**
(DOC)
