# Supplementary material for: Metabolic Engineering of the Phenylpropanoid Pathway Enhances the Antioxidant Capacity of Saussurea involucrata
Source: PLoS One. 2013 Aug 14;8(8):e70665. doi: 10.1371/journal.pone.0070665 (PMC3743766; doi:10.1371/journal.pone.0070665)
Supplement: Figure S1 — Survival rate of S. involucrata calli on medium supplemented with different concentrations of Hygromycin. (DOC) [file pone.0070665.s001.doc]

**Figure S1** Survival rate of *S. involucrata* calli on medium supplemented with different concentrations of Hygromycin.
