# Supplementary material for: Metabolic Engineering of the Phenylpropanoid Pathway Enhances the Antioxidant Capacity of Saussurea involucrata
Source: PLoS One. 2013 Aug 14;8(8):e70665. doi: 10.1371/journal.pone.0070665 (PMC3743766; doi:10.1371/journal.pone.0070665)
Supplement: Figure S2 — PCR assay of selective marker gus gene in transgenic plants. (DOC) [file pone.0070665.s002.doc]

**Figure S2** PCR assay of selective marker *gus* gene in transgenic *S. involucrata* plants (M: DL2000 marker, CT: Wild type, 1: pCAMBIA1301 plasmid, 2-23: different lines of transgenic callus).
