# Supplementary material for: Metabolic Engineering of the Phenylpropanoid Pathway Enhances the Antioxidant Capacity of Saussurea involucrata
Source: PLoS One. 2013 Aug 14;8(8):e70665. doi: 10.1371/journal.pone.0070665 (PMC3743766; doi:10.1371/journal.pone.0070665)
Supplement: Figure S3 — PCR assay of PAP1 and Lc in transgenic plants. (DOC) [file pone.0070665.s003.doc]

**Figure S3** PCR assay of *PAP1* and *Lc* genes in transgenic plants. (a) transgenic calli co-expressing *PAP1* and *Lc* (C1-C6). (b) transgenic shoots co-expressing *PAP1* and *Lc* (P1-P3). (c) transgenic calli expressing *PAP1* (left panel) or *Lc* (right panel) alone. M, DL2000 marker; CT, Wild type; 1, PCR product of *PAP1*; 2, PCR product of *Lc*.
