# Supplementary material for: Metabolic Engineering of the Phenylpropanoid Pathway Enhances the Antioxidant Capacity of Saussurea involucrata
Source: PLoS One. 2013 Aug 14;8(8):e70665. doi: 10.1371/journal.pone.0070665 (PMC3743766; doi:10.1371/journal.pone.0070665)
Supplement: Table S1 — KEGG pathway assignment in S. involucrata . (DOC) [file pone.0070665.s004.doc]

**Table S1 KEGG pathway assignment in *S. involucrata.***

| **No.** | **Pathway** | **Count** | **Percentage**  **(%)** | **Pathway ID** |
| --- | --- | --- | --- | --- |
| 1 | [Metabolic pathways](#RANGE!gene1) | 4109 | 22.38 | ko01100 |
| 2 | [Plant-pathogen interaction](#RANGE!gene2) | 1324 | 7.21 | ko04626 |
| 3 | [Spliceosome](#RANGE!gene3) | 1108 | 6.03 | ko03040 |
| 4 | [Biosynthesis of plant hormones](#RANGE!gene4) | 906 | 4.93 | ko01070 |
| 5 | [Biosynthesis of phenylpropanoids](#RANGE!gene5) | 736 | 4.01 | ko01061 |
| 6 | [Starch and sucrose metabolism](#RANGE!gene6) | 618 | 3.37 | ko00500 |
| 7 | [Biosynthesis of terpenoids and steroids](#RANGE!gene7) | 591 | 3.22 | ko01062 |
| 8 | [Ribosome](#RANGE!gene8) | 571 | 3.11 | ko03010 |
| 9 | [Biosynthesis of alkaloids derived from shikimate pathway](#RANGE!gene9) | 514 | 2.80 | ko01063 |
| 10 | [Biosynthesis of alkaloids derived from ornithine, lysine and nicotinic acid](#RANGE!gene10) | 496 | 2.70 | ko01064 |
| 11 | [Biosynthesis of alkaloids derived from terpenoid and polyketide](#RANGE!gene11) | 464 | 2.53 | ko01066 |
| 12 | [Ubiquitin mediated proteolysis](#RANGE!gene12) | 454 | 2.47 | ko04120 |
| 13 | [Biosynthesis of alkaloids derived from histidine and purine](#RANGE!gene13) | 435 | 2.37 | ko01065 |
| 14 | [Endocytosis](#RANGE!gene14) | 433 | 2.36 | ko04144 |
| 15 | [Purine metabolism](#RANGE!gene15) | 427 | 2.33 | ko00230 |
| 16 | [Phenylpropanoid biosynthesis](#RANGE!gene16) | 411 | 2.24 | ko00940 |
| 17 | [Pyrimidine metabolism](#RANGE!gene17) | 348 | 1.90 | ko00240 |
| 18 | [Oxidative phosphorylation](#RANGE!gene18) | 340 | 1.85 | ko00190 |
| 19 | [Glycolysis / Gluconeogenesis](#RANGE!gene19) | 334 | 1.82 | ko00010 |
| 20 | [RNA degradation](#RANGE!gene20) | 322 | 1.75 | ko03018 |
| 21 | [Cysteine and methionine metabolism](#RANGE!gene21) | 271 | 1.48 | ko00270 |
| 22 | [Amino sugar and nucleotide sugar metabolism](#RANGE!gene22) | 262 | 1.43 | ko00520 |
| 23 | [Peroxisome](#RANGE!gene23) | 259 | 1.41 | ko04146 |
| 24 | [Glycerophospholipid metabolism](#RANGE!gene24) | 251 | 1.37 | ko00564 |
| 25 | [Limonene and pinene degradation](#RANGE!gene25) | 247 | 1.35 | ko00903 |
| 26 | [Pyruvate metabolism](#RANGE!gene26) | 235 | 1.28 | ko00620 |
| 27 | [Stilbenoid, diarylheptanoid and gingerol biosynthesis](#RANGE!gene27) | 232 | 1.26 | ko00945 |
| 28 | [Circadian rhythm - plant](#RANGE!gene28) | 214 | 1.17 | ko04712 |
| 29 | [ABC transporters](#RANGE!gene29) | 202 | 1.10 | ko02010 |
| 30 | [Flavonoid biosynthesis](#RANGE!gene30) | 198 | 1.08 | ko00941 |
| 31 | [RNA polymerase](#RANGE!gene31) | 198 | 1.08 | ko03020 |
| 32 | [Carbon fixation in photosynthetic organisms](#RANGE!gene32) | 193 | 1.05 | ko00710 |
| 33 | [Nucleotide excision repair](#RANGE!gene33) | 180 | 0.98 | ko03420 |
| 34 | [Cyanoamino acid metabolism](#RANGE!gene34) | 170 | 0.93 | ko00460 |
| 35 | [Galactose metabolism](#RANGE!gene35) | 165 | 0.90 | ko00052 |
| 36 | [Nitrogen metabolism](#RANGE!gene36) | 162 | 0.88 | ko00910 |
| 37 | [Arginine and proline metabolism](#RANGE!gene37) | 160 | 0.87 | ko00330 |
| 38 | [Fatty acid metabolism](#RANGE!gene38) | 159 | 0.87 | ko00071 |
| 39 | [Citrate cycle (TCA cycle)](#RANGE!gene39) | 159 | 0.87 | ko00020 |
| 40 | [Phosphatidylinositol signaling system](#RANGE!gene40) | 158 | 0.86 | ko04070 |
| 41 | [Pentose and glucuronate interconversions](#RANGE!gene41) | 158 | 0.86 | ko00040 |
| 42 | [Fructose and mannose metabolism](#RANGE!gene42) | 157 | 0.86 | ko00051 |
| 43 | [Tryptophan metabolism](#RANGE!gene43) | 156 | 0.85 | ko00380 |
| 44 | [Glutathione metabolism](#RANGE!gene44) | 150 | 0.82 | ko00480 |
| 45 | [Proteasome](#RANGE!gene45) | 149 | 0.81 | ko03050 |
| 46 | [alpha-Linolenic acid metabolism](#RANGE!gene46) | 147 | 0.80 | ko00592 |
| 47 | [Glycerolipid metabolism](#RANGE!gene47) | 146 | 0.80 | ko00561 |
| 48 | [Zeatin biosynthesis](#RANGE!gene48) | 144 | 0.78 | ko00908 |
| 49 | [Ascorbate and aldarate metabolism](#RANGE!gene49) | 142 | 0.77 | ko00053 |
| 50 | [Aminoacyl-tRNA biosynthesis](#RANGE!gene50) | 142 | 0.77 | ko00970 |
| 51 | [Alanine, aspartate and glutamate metabolism](#RANGE!gene51) | 140 | 0.76 | ko00250 |
| 52 | [Biosynthesis of unsaturated fatty acids](#RANGE!gene52) | 137 | 0.75 | ko01040 |
| 53 | [Inositol phosphate metabolism](#RANGE!gene53) | 134 | 0.73 | ko00562 |
| 54 | [Phenylalanine metabolism](#RANGE!gene54) | 131 | 0.71 | ko00360 |
| 55 | [Base excision repair](#RANGE!gene55) | 129 | 0.70 | ko03410 |
| 56 | [Propanoate metabolism](#RANGE!gene56) | 125 | 0.68 | ko00640 |
| 57 | [Valine, leucine and isoleucine degradation](#RANGE!gene57) | 125 | 0.68 | ko00280 |
| 58 | [Butanoate metabolism](#RANGE!gene58) | 124 | 0.68 | ko00650 |
| 59 | [Tyrosine metabolism](#RANGE!gene59) | 123 | 0.67 | ko00350 |
| 60 | [Ether lipid metabolism](#RANGE!gene60) | 120 | 0.65 | ko00565 |
| 61 | [Basal transcription factors](#RANGE!gene61) | 120 | 0.65 | ko03022 |
| 62 | [DNA replication](#RANGE!gene62) | 119 | 0.65 | ko03030 |
| 63 | [Lysine degradation](#RANGE!gene63) | 112 | 0.61 | ko00310 |
| 64 | [N-Glycan biosynthesis](#RANGE!gene64) | 109 | 0.59 | ko00510 |
| 65 | [Protein export](#RANGE!gene65) | 105 | 0.57 | ko03060 |
| 66 | [Glycine, serine and threonine metabolism](#RANGE!gene66) | 105 | 0.57 | ko00260 |
| 67 | [Photosynthesis](#RANGE!gene67) | 101 | 0.55 | ko00195 |
| 68 | [Carotenoid biosynthesis](#RANGE!gene68) | 101 | 0.55 | ko00906 |
| 69 | [Pentose phosphate pathway](#RANGE!gene69) | 100 | 0.54 | ko00030 |
| 70 | [SNARE interactions in vesicular transport](#RANGE!gene70) | 99 | 0.54 | ko04130 |
| 71 | [Homologous recombination](#RANGE!gene71) | 98 | 0.53 | ko03440 |
| 72 | [Mismatch repair](#RANGE!gene72) | 98 | 0.53 | ko03430 |
| 73 | [Methane metabolism](#RANGE!gene73) | 98 | 0.53 | ko00680 |
| 74 | [Metabolism of xenobiotics by cytochrome P450](#RANGE!gene74) | 98 | 0.53 | ko00980 |
| 75 | [Porphyrin and chlorophyll metabolism](#RANGE!gene75) | 89 | 0.48 | ko00860 |
| 76 | [Valine, leucine and isoleucine biosynthesis](#RANGE!gene76) | 89 | 0.48 | ko00290 |
| 77 | [Fatty acid biosynthesis](#RANGE!gene77) | 88 | 0.48 | ko00061 |
| 78 | [Linoleic acid metabolism](#RANGE!gene78) | 87 | 0.47 | ko00591 |
| 79 | [Terpenoid backbone biosynthesis](#RANGE!gene79) | 87 | 0.47 | ko00900 |
| 80 | [Ubiquinone and other terpenoid-quinone biosynthesis](#RANGE!gene80) | 87 | 0.47 | ko00130 |
| 81 | [Steroid biosynthesis](#RANGE!gene81) | 86 | 0.47 | ko00100 |
| 82 | [beta-Alanine metabolism](#RANGE!gene82) | 86 | 0.47 | ko00410 |
| 83 | [Regulation of autophagy](#RANGE!gene83) | 86 | 0.47 | ko04140 |
| 84 | [Sphingolipid metabolism](#RANGE!gene84) | 78 | 0.42 | ko00600 |
| 85 | [Phenylalanine, tyrosine and tryptophan biosynthesis](#RANGE!gene85) | 75 | 0.41 | ko00400 |
| 86 | [Other glycan degradation](#RANGE!gene86) | 73 | 0.40 | ko00511 |
| 87 | [Flavone and flavonol biosynthesis](#RANGE!gene87) | 73 | 0.40 | ko00944 |
| 88 | [Selenoamino acid metabolism](#RANGE!gene88) | 72 | 0.39 | ko00450 |
| 89 | [Glyoxylate and dicarboxylate metabolism](#RANGE!gene89) | 70 | 0.38 | ko00630 |
| 90 | [Natural killer cell mediated cytotoxicity](#RANGE!gene90) | 66 | 0.36 | ko04650 |
| 91 | [Histidine metabolism](#RANGE!gene91) | 62 | 0.34 | ko00340 |
| 92 | [Isoquinoline alkaloid biosynthesis](#RANGE!gene92) | 57 | 0.31 | ko00950 |
| 93 | [Diterpenoid biosynthesis](#RANGE!gene93) | 51 | 0.28 | ko00904 |
| 94 | [Benzoxazinoid biosynthesis](#RANGE!gene94) | 50 | 0.27 | ko00402 |
| 95 | [Tropane, piperidine and pyridine alkaloid biosynthesis](#RANGE!gene95) | 46 | 0.25 | ko00960 |
| 96 | [Glycosaminoglycan degradation](#RANGE!gene96) | 45 | 0.25 | ko00531 |
| 97 | [Pantothenate and CoA biosynthesis](#RANGE!gene97) | 44 | 0.24 | ko00770 |
| 98 | [Glucosinolate biosynthesis](#RANGE!gene98) | 39 | 0.21 | ko00966 |
| 99 | [Nicotinate and nicotinamide metabolism](#RANGE!gene99) | 39 | 0.21 | ko00760 |
| 100 | [Lysine biosynthesis](#RANGE!gene100) | 38 | 0.21 | ko00300 |
| 101 | [Sulfur metabolism](#RANGE!gene101) | 37 | 0.20 | ko00920 |
| 102 | [Arachidonic acid metabolism](#RANGE!gene102) | 36 | 0.20 | ko00590 |
| 103 | [Non-homologous end-joining](#RANGE!gene103) | 36 | 0.20 | ko03450 |
| 104 | [Glycosphingolipid biosynthesis - ganglio series](#RANGE!gene104) | 31 | 0.17 | ko00604 |
| 105 | [Polyketide sugar unit biosynthesis](#RANGE!gene105) | 29 | 0.16 | ko00523 |
| 106 | [Photosynthesis - antenna proteins](#RANGE!gene106) | 28 | 0.15 | ko00196 |
| 107 | [Glycosylphosphatidylinositol(GPI)-anchor biosynthesis](#RANGE!gene107) | 26 | 0.14 | ko00563 |
| 108 | [One carbon pool by folate](#RANGE!gene108) | 26 | 0.14 | ko00670 |
| 109 | [Folate biosynthesis](#RANGE!gene109) | 23 | 0.13 | ko00790 |
| 110 | [Indole alkaloid biosynthesis](#RANGE!gene110) | 23 | 0.13 | ko00901 |
| 111 | [Riboflavin metabolism](#RANGE!gene111) | 23 | 0.13 | ko00740 |
| 112 | [Anthocyanin biosynthesis](#RANGE!gene112) | 21 | 0.11 | ko00942 |
| 113 | [Thiamine metabolism](#RANGE!gene113) | 20 | 0.11 | ko00730 |
| 114 | [Synthesis and degradation of ketone bodies](#RANGE!gene114) | 18 | 0.10 | ko00072 |
| 115 | [Brassinosteroid biosynthesis](#RANGE!gene115) | 17 | 0.09 | ko00905 |
| 116 | [Monoterpenoid biosynthesis](#RANGE!gene116) | 14 | 0.08 | ko00902 |
| 117 | [Vitamin B6 metabolism](#RANGE!gene117) | 13 | 0.07 | ko00750 |
| 118 | [Glycosphingolipid biosynthesis - globo series](#RANGE!gene118) | 12 | 0.07 | ko00603 |
| 119 | [Caffeine metabolism](#RANGE!gene119) | 12 | 0.07 | ko00232 |
| 120 | [C5-Branched dibasic acid metabolism](#RANGE!gene120) | 10 | 0.05 | ko00660 |
| 121 | [Betalain biosynthesis](#RANGE!gene121) | 9 | 0.05 | ko00965 |
| 122 | [Lipoic acid metabolism](#RANGE!gene122) | 8 | 0.04 | ko00785 |
| 123 | [Taurine and hypotaurine metabolism](#RANGE!gene123) | 7 | 0.04 | ko00430 |
| 124 | [Biotin metabolism](#RANGE!gene124) | 7 | 0.04 | ko00780 |
| 125 | [Fatty acid elongation in mitochondria](#RANGE!gene125) | 6 | 0.03 | ko00062 |
