# Supplementary material for: Metabolic Engineering of the Phenylpropanoid Pathway Enhances the Antioxidant Capacity of Saussurea involucrata
Source: PLoS One. 2013 Aug 14;8(8):e70665. doi: 10.1371/journal.pone.0070665 (PMC3743766; doi:10.1371/journal.pone.0070665)
Supplement: Table S2 — Functional annotations of some key unigenes involved in the phenylpropanoid biosynthesis. (DOC) [file pone.0070665.s005.doc]

**Table S2 Functional annotations of some key unigenes involved in the phenylpropanoid biosynthesis.**

| Gene ID | Gene  Length | Nr- ID | Nr-E-value | Swissprot  ID | Swissprot  E-value | KEGG  E-value | KEGG-Gene | Abbreviation |
| --- | --- | --- | --- | --- | --- | --- | --- | --- |
| Phenylalanine ammonia-lyase | | | | | | | | |
| Unigene18921 | 1239 | gi|27436243 | 0 | sp|P45729 | 0 | 0 | vvi:100263160 | PAL1 |
| Unigene20928 | 1290 | gi|1171998 | 0 | sp|P45726 | 0 | 0 | vvi:100266593 | PAL2 |
| *trans*-Cinnamate 4-monooxygenase | | | | | | | | |
| Unigene12874 | 1674 | gi|417863 | 0 | sp|Q04468 | 0 | 0 | pop:POPTR_823837 | C4H1 |
| Unigene54861 | 944 | gi|417863 | 1.00E-172 | sp|Q04468 | 3.00E-172 | 2.00E-167 | rcu:RCOM_1164350 | C4H2 |
| 4-Coumarate-CoA ligase | | | | | | | | |
| Unigene24861 | 1682 | gi|294516938 | 0 | sp|P31687 | 0 | 0 | vvi:100245991 | 4CL1 |
| Unigene55623 | 1268 | gi|1237183 | 0 | sp|O24145 | 0 | 0 | vvi:100254698 | 4CL2 |
| *p*-Coumarate 3-hydroxylase | | | | | | | | |
| Unigene56048 | 1811 | gi|85068608 | 0 | sp|O48922 | 0 | 0 | pop:POPTR_685839 | C3'H1 |
| Unigene2390 | 1441 | gi|22573441 | 0 | sp|O48922 | 0 | 0 | pop:POPTR_685839 | C3'H2 |
| Ferulate-5-hydroxylase | | | | | | | | |
| Unigene54860 | 944 | gi|46403211 | 1.00E-136 | sp|Q42600 | 3.00E-131 | 3.00E-132 | ath:AT4G36220 | F5H |
| Cinnamoyl-CoA reductase | | | | | | | | |
| Unigene55560 | 1221 | gi|82941439 | 1.00E-169 | sp|Q9XES5 | 1.00E-61 | 7.00E-143 | ath:AT1G80820 | CCR |
| Cinnamyl-alcohol dehydrogenase | | | | | | | | |
| Unigene51321 | 525 | gi|206236379 | 1.00E-55 | sp|Q2KNL6 | 1.00E-51 | 3.00E-47 | ath:AT4G37970 | CAD1 |
| Unigene55296 | 1088 | gi|75333650 | 1.00E-146 | sp|Q9CAI3 | 4.00E-146 | 9.00E-89 | ath:AT2G21890 | CAD2 |
| Coniferyl-aldehyde dehydrogenase | | | | | | | | |
| Unigene299 | 479 | gi|18404212 | 2.00E-44 | sp|Q56YU0 | 9.00E-45 | 9.00E-46 | ath:AT3G24503 | ALDH |
| Flavone synthase II | | |  |  |  |  |  |  |
| Unigene55906 | 1545 | gi|171906244 | 0 | sp|P93149 | 8.00E-159 | 5.00E-105 | ath:AT4G15350 | FNSII |
| Flavonol synthase | |  |  |  |  |  |  |  |
| Unigene53321 | 684 | gi|126116624 | 1.00E-112 | sp|Q9M547 | 5.00E-99 | 1.00E-89 | ath:AT5G08640 | FLS |
| beta-Glucosidase | |  |  |  |  |  |  |  |
| Unigene24695 | 1211 | gi|75296458 | 1.00E-124 | sp|Q7XSK0 | 2.00E-124 | 8.00E-124 | pop:POPTR_856874 | BGLU |
| Myb transcription factor (AtMyb4) | | | | | | | | |
| Unigene18654 | 970 | gi|225428007 | 6.00E-91 | sp|P81393 | 3.00E-81 | 3.00E-75 | ath:AT4G38620 | MYB1 |
| Myb transcription factor (AtMyb 90) | | | | | | | | |
| Unigene52423 | 597 | gi|40643886 | 1.00E-64 | sp|Q9ZTC3 | 2.00E-34 | 2.00E-35 | ath:AT1G66390 | MYB2 |
| Myb transcription factor (AtMyb 12) | | | | | | | | |
| Unigene11699 | 1023 | gi|258558776 | 8.00E-61 | sp|O22264 | 2.00E-59 | 2.00E-60 | ath:AT2G47460 | MYB3 |
| bHLH transcription factor (TT8) | | | | | | | | |
| Unigene53148 | 663 | gi|184161316 | 5.00E-45 | sp|Q9FT81 | 3.00E-25 | 3.00E-09 | sbi:SORBI_01g028230 | bHLH1 |
| Wd repeat protein (AtTTG1) | | | | | | | | |
| Unigene1831 | 483 | gi|224581456 | 3.00E-86 | sp|Q9XGN1 | 3.00E-77 | 3.00E-64 | ppp:PHYPADRAFT_132054 | WDR1 |
| Chalcone synthase | | | | | | | | |
| Unigene54614 | 886 | gi|85542655 | 1.00E-145 | sp|Q9FUB7 | 1.00E-132 | 2.00E-132 | pop:POPTR_554829 | CHS1 |
| Unigene55749 | 1364 | gi|3915638 | 0 | sp|P48385 | 0 | 0 | vvi:100263443 | CHS2 |
| Unigene55521 | 1197 | gi|1345793 | 0 | sp|P48392 | 0 | 0 | vvi:100263443 | CHS3 |
| Unigene55563 | 1222 | gi|2326772 | 1.00E-157 | sp|P08894 | 9.00E-72 | 2.00E-73 | vvi:100266849 | CHS4 |
| Unigene55618 | 1262 | gi|224078067 | 1.00E-176 | sp|O04111 | 1.00E-81 | 4.00E-82 | pop:POPTR_814871 | CHS5 |
| Chalcone isomerase | | | | | | | | |
| Unigene53852 | 750 | gi|75156641 | 1.00E-123 | sp|Q8LKP9 | 2.00E-123 | 3.00E-75 | ath:AT3G55120 | CHI |
| Flavanone-3-beta-hydroxylase | | | | | | | | |
| Unigene54561 | 874 | gi|171906248 | 1.00E-143 | sp|Q05963 | 1.00E-141 | 4.00E-129 | ath:AT3G51240 | F3H |
| Dihydroflavonol 4-reductase | | | | | | | | |
| Unigene54955 | 967 | gi|148628025 | 0 | sp|P51105 | 1.00E-159 | 3.00E-136 | vvi:100233141 | DFR1 |
| Unigene15721 | 998 | gi|124359568 | 7.00E-85 | sp|Q9SEV0 | 9.00E-32 | 8.00E-33 | ath:AT1G61720 | DFR2 |
| Unigene54992 | 980 | gi|76559894 | 1.00E-133 | sp|P5257 | 3.00E-121 | 3.00E-116 | ath:AT1G75280 | DFR3 |
| Unigene55375 | 1131 | gi|149349541 | 1.00E-142 | sp|P52577 | 4.00E-134 | 4.00E-135 | ath:AT1G75280 | DFR4 |
| Anthocyanidin synthase | | | | | | | | |
| Unigene16079 | 1211 | gi|44889072 | 1.00E-164 | sp|P51092 | 6.00E-137 | 2.00E-132 | ath:AT4G22880 | ANS |
| Flavonoid 3'-hydroxylase | | | | | | | | |
| Unigene56029 | 1771 | gi|224815364 | 0 | sp|Q9SBQ9 | 0 | 0 | vvi:100232999 | F3’H |
| Flavonoid 3',5'-hydroxylase | | | | | | | | |
| Unigene53432 | 695 | gi|224131390 | 2.00E-73 | sp|P37120 | 3.00E-46 | 1.00E-47 | pop:POPTR_54623 | F3’5’H |
| Unigene55750 | 1365 | gi|14423327 | 0 | sp|Q9SBQ9 | 1.00E-106 | 2.00E-108 | vvi:100232999 | F3’5’H |
| Glucosyltransferase |  |  |  |  |  |  |  |  |
| Unigene54571 | 876 | gi|133874216 | 2.00E-90 | sp|Q9SBQ8 | 8.00E-74 | 3.00E-66 | vvi:100233099 | UGT1 |
| Unigene55355 | 1119 | gi|133874216 | 1.00E-96 | sp|P51094 | 4.00E-73 | 4.00E-74 | vvi:100233099 | UGT2 |
| Unigene24251 | 1004 | gi|2501494 | 1E-78 | sp|Q40287 | 8E-79 | 4E-79 | vvi:100265092 | UGT3 |
| Caffeoyl-CoA methyltransferase | | | | | | | | |
| Unigene 54824 | 934 | gi|207059702 | 1.00E-137 | sp|Q41720 | 2.00E-129 | 4.00E-124 | ath:AT4G34050 | CCOMT1 |
| Unigene 25273 | 850 | gi|207060911 | 1.00E-133 | sp|Q43095 | 1.00E-127 | 4.00E-126 | ath:AT4G34050 | CCOMT2 |
| Unigene 49796 | 454 | gi|207061309 | 5.00E-49 | sp|O65162 | 3.00E-49 | 3.00E-47 | ath:AT4G34050 | CCOMT3 |
| Flavonoid *O*-methyltransferase | | | | | | | | |
| Unigene 51655 | 545 | gi|29839290 | 1.00E-93 | sp|Q43239 | 5.00E-85 | 4.00E-78 | ath:AT5G54160 | FOMT1 |
| Unigene 19256 | 619 | gi|207060911 | 1.00E-107 | sp|Q43239 | 1.00E-107 | 2.00E-99 | ath:AT5G54160 | FOMT2 |
| Anthocyanidin 5-*O*-glucoside-6"-*O*-malonyltransferase | | | | | | | |  |
| Unigene 24950 | 1138 | gi|146387237 | 1.00E-130 | sp|Q9ZWR8 | 4.00E-53 | 4.00E-17 | pop:POPTR_554899 | MAT |
