# Supplementary material for: Metabolic Engineering of the Phenylpropanoid Pathway Enhances the Antioxidant Capacity of Saussurea involucrata
Source: PLoS One. 2013 Aug 14;8(8):e70665. doi: 10.1371/journal.pone.0070665 (PMC3743766; doi:10.1371/journal.pone.0070665)
Supplement: Table S3 — Growth and induction data of immature embryo calli. (DOC) [file pone.0070665.s006.doc]

**Table S3 Growth and induction data of immature embryo calli.**

| Culture medium | 2,4-D | 6-BA | Induction rate (30 d) | Growth rate | Growth status |
| --- | --- | --- | --- | --- | --- |
| MS | 1 | - | 12.7% | * | ++ |
|  | 2 | - | 16.3% | ** | ++ |
|  | 3 | - | 36% | ** | ++ |
|  | 4 | - | 34% | *** | + |
|  | 5 | - | 33.7% | **** | + |
|  | 3 | 0.2 | 33.3% | *** | ++++ |
| NB | 3 | 0 | 42.7% | *** | ++ |
|  | 3 | 0.2 | 39.7% | *** | ++++ |

Induction rate: The number of immature embryogenic callus after 30-day induction.* Growth rate: The growth rate of subculture, ****: Fast, *: Slowest; + Growth status: The growth status on subculture, ++++: Optimum growth (incompact, graininess and yellow callus), +: Worst growth (humid and brown callus). The abbreviations are: 2,4-D, 2,4-Dichlorophenoxyacetic acid; 6-BA: 6-Benzylaminopurine.
