# Supplementary material for: Metabolic Engineering of the Phenylpropanoid Pathway Enhances the Antioxidant Capacity of Saussurea involucrata
Source: PLoS One. 2013 Aug 14;8(8):e70665. doi: 10.1371/journal.pone.0070665 (PMC3743766; doi:10.1371/journal.pone.0070665)
Supplement: Table S4 — Primers used in this study. (DOC) [file pone.0070665.s007.doc]

**Table S4 Primers used in this study.**

| **Gene name** | **Forward primer (5’-3’)** | **Reverse primer (5’-3’)** |
| --- | --- | --- |
| **Gene-specific primer sets for transgenic verification by PCR** | | |
| *GUS* | TGCTGTCGGCTTTAACCTCT | GGCACAGCACATCAAAGAGA |
| *PAP1* | AATTGCTGGAAGATTACCTGG | CCATCGAAAAGACTCCAAAGT |
| *Lc* | GACGCAAGCCTGGAGCACATC | GGCCACCACTTTCTTCAGCAATC |
| **Primer sets for quantitative real-time PCR** | | |
| *PAL1* | GGTGAAGGCGAGTAGTGATTGG | GCTGAATGTGGAAGGGTGTGA |
| *C4H* | TGACACCAAACTCGGACCAG | ACCAGGCGTTGACCAAGAT |
| *4CL* | GACGAGTGTAGCACAGCAAGT | ACGCACATCAGCACATCCT |
| *C3H1* | GCCGCCATCGTCTTCATTC | CGTAGGTTCTCGCCCATTCA |
| *C3H2* | CGGAGGAACCAAGCACCAT | CCATCGCCCACTCAACAGA |
| *F5H* | GTCAACGCATACGCCATCAAC | CGCCATTTCCATCGCATACAAC |
| *CCR* | GACGAGGCTAAGGCGAGAG | TGGACCGAATTGGCGTAGG |
| *CAD1* | CCATTGCTTAGTGTGCTCAAACC | AGGGAGTTGGCGACATCAATC |
| *CAD2* | AGAGTGTGAGTATTGCGATGAAGG | GCTGCCAAAGATAACGGGTAGT |
| *CHS* | GACACGCTTCGGCTACAGAT | AATGGACGCACTATGGAGGAC |
| *CHI* | CATTCCGTCACTCGCCGTTA | TGTAGGTCCCTTGTGCTTTCC |
| *F3H* | ACTACCCGAAATGCCCTCAG | TCTTGCCACCATCACGAGTC |
| *DFR1* | TCCATTCATCACTCCTTCGTTCC | TCTTCCTTTGGCTTTAGGGTTCTC |
| *DFR3* | ATCCTACTTTCCTCCTCGTCAGA | TCTACCTGCTTTATCGCCTTCAC |
| *DFR4* | CGGCTACTTCCTACCTACATTGG | TTCTCGGATCATCTACGGCTTTG |
| *ANS* | ACAACGCCCGACGATTACAT | CCCGAGAGATAGAACCGAGAGT |
| *F3’H* | GGATGACGCCGATGGAGAA | CGATGGGTGGAGCCTGAAG |
| *F3’5’H1* | CGACACTTCCGCTGATGGT | GGTTCCCGCACATATCCTCC |
| *F3’5’H2* | GTTGAATGGGCGATTGCTGAA | CCCGATGTAAGGTAGGTTAGGC |
| *UGT1* | TCCGATTGCCACAACATACGA | TTCATTTCCTCCGCCATATCTCC |
| *UGT2* | CTCCGATTTCAACTGCCACAACCT | CACCTTAACACCCTTCCTCAA |
| *UGT3* | GCCATCCGTACCATCATATCCTC | CAACAAATTGACCCACCACTTCC |
| *CCoAM1* | AAGGTCCTGCTCTTCCTGTTC | CGATCCGTTCCAAAGGGTGTTA |
| *CCoAM2* | GAGAGGTCACGGCGAAACA | CGGTAGCAAGGAGGGAGTAAC |
| *CCoAM3* | GAACCAACCGAACACAAAGGC | ACGCAATCATCTGACCAGCAT |
| *FOMT1* | TGGTGGATGTTGGTGGTGG | TGAGCGGTTGCGTCTTGA |
| *FOMT2* | CGCTTCGCTCAAGGGTATCA | GGCAATGTGCATCACTCCAATC |
| *MYB* | GCTGTAGGTTGCGATGGTTGA | TGCGGTTCTTCCTGGTATTCTTC |
| *WDR* | CACCGTTGTTGAGATTGGCTTG | CGCATTCACACTCCCTCTATGTC |
| *Lc* | GTCACTGCTTCCGTCCATTCA | ACCTCCTTCCTCACACTCTCAT |
| *PAP1* | AGGCTTCTAGGGAATAGGTGGT | TGTAGGAATGGGCGTAATGTCTC |
| *GAPDH* | CCAAGAAGACATTCGCTGAGGA | CGCACACCGACAAGATACCA |

All primers were designed based on the unigene sequences in *S. involucrata*. The abbreviations are the same as listed in Table S1*.*
