# Supplementary material for: Metabolic Engineering of the Phenylpropanoid Pathway Enhances the Antioxidant Capacity of Saussurea involucrata
Source: PLoS One. 2013 Aug 14;8(8):e70665. doi: 10.1371/journal.pone.0070665 (PMC3743766; doi:10.1371/journal.pone.0070665)
Supplement: Table S5 — Identification of phenylpropanoid compounds in S. involucrata . (DOC) [file pone.0070665.s008.doc]

**Table S5 Identification of phenylpropanoid compounds in *S. involucrata*.**

|  | Compounds  name | Retention  time (min) | MS (Positive Ionization) | | | | |  | MS (Negative Ionization) | | | UV | E_440_/  E_vis-max_ | References |
| --- | --- | --- | --- | --- | --- | --- | --- | --- | --- | --- | --- | --- | --- | --- |
|  |  |  | [M+H]^+^  (*m/z*) | Molecular  structure (+H) | Aglycone  (*m/z*) | Molecular structure  of aglycone (+H) | Other fragment  (*m/z*) |  | [M-H]^–^  (m/z) | Aglycone  (*m/z*) | Other fragment  (*m/z*) |  |  |  |
| 1 | 3-caffeoyl quinic acid ^#^ | 1.064 | 355.0509 | C_16_H_19_O_9_ |  |  |  |  | 353.0798 |  | 191.0142,  179.0125,  172.9449 | 222.1, 325.1 |  | Clifford *et al.* 2003 |
| 2 | Chlorogenic acid^*^ | 2.107 | 355.1030 | C_16_H_19_O_9_ |  |  | 163.0395 |  | 353.0832 |  | 191.0436,  179.0286 | 218.9, 325.1 |  | Qiu *et al*. 2010;  Yi *et al*. 2009 |
| 3 | 4-caffeoylquinic acid ^#^ | 2.798 |  | C_16_H_19_O_9_ |  |  |  |  | 353.0852 |  | 173.0410,  179.0276,  191.0213 | 239.7, 325.7 |  | Clifford *et al*. 2003 |
| 4 | Syringin^*^ | 2.828 | 395.1290  ([M+Na]^+^) | C_17_H_24_O_9_Na | 211.0925 | C_11_H_15_O_4_ |  |  |  |  |  | 222.0, 264.1 |  | Qiu *et al*. 2010;  Yi *et al*. 2009;  Yu *et al*. 2006 |
| 5 | Cyanidin 3-*O*-glucoside^*^ | 3.879 | 449.1062 | C_21_H_21_O_11_ | 287.0556 | C_15_H_11_O_6_ |  |  |  |  |  | 280.8, 515.8 | 31.38% | Based on standard |
| 6 | Cyanidin 3-malonylglucoside ^#^ | 9.467 | 535.1085 | C_24_H_23_O_14_ | 287.0547 | C_15_H_11_O_6_ | 449.0598 |  |  |  |  | 281.3, 517.2 | 30.90% | Saito *et al*. 1988 |
| 7 | Rutin^*^ | 10.093 | 611.1602 | C_27_H_31_O_16_ | 303.0502 | C_15_H_11_O_7_ | 465.0986 |  | 609.1467 |  |  | 255.6, 353.7 |  | Qiu *et al*. 2010;  Fu *et al*. 2006 |
| 8 | Quercetin 3-*O*-glucoside ^#^ | 10.150 | 465.1042 | C_21_H_21_O_12_ | 303.047 |  |  |  | 463.0753 | 301.0323 |  | 255.6, 352.5 |  | Hokkanen *et al*.2009 |
| 9 | Cyanidin 3-*O*-malonylglucoside ^#^ | 10.353 | 535.1090 | C_24_H_23_O_14_ | 287.0549 | C_15_H_11_O_6_ |  |  |  |  |  | 281.5, 518.7 |  | Saito *et al*. 1988 |
| 10 | Luteolin 7-*O*-neohesperidoside ^#^ | 10.806 | 595.1620 | C_27_H_31_O_15_ | 287.055 | C_15_H_11_O_6_ | 449.1099 |  | 593.1531 | 284.992 | 477.1092 | n.a. |  | Yi *et al*. 2009 |
| 11 | Cynarine^*^ | 10.944 | 517.1348 | C_25_H_25_O_12_ |  |  | 499.1227,  163.0397 |  | 515.0986 |  | 191.0486 | 219.8, 243.4,  328.9 |  | Qiu *et al*. 2010;  Yi *et al*. 2009;  Fu *et al*. 2006 |
| 12 | Cyanidin-malonylglucoside ^#^ | 11.447 | 535.1093 | C_24_H_23_O_14_ | 287.058 | C_15_H_11_O_6_ |  |  |  |  |  | n.a. | n.a. | Saito *et al*. 1988 |
| 13 | Chrysoeriol 7-*O*-glucoside ^#^ | 11.576 | 463.1249 | C_22_H_23_O_11_ | 301.0708 | C_16_H_13_O_6_ |  |  | 461.1011 | 299.1154 |  | 252.5, 347.5 |  | Li *et al*. 2009;  Lin *et al*. 2010 |
| 14 | Hispidulin 7-*O*-glucoside ^#^ | 11.693 | 463.1231 | C_22_H_23_O_11_ | 301.067 | C_16_H_13_O_6_ |  |  | 461.1124 |  |  | 273.4, 333.2 |  | Yi *et al*. 2009 |
| 15 | Arctiin^*^ | 13.572 | 557.2012  ([M+Na]^+^) | C_27_H_34_O_11_Na | 373.1624 | C_21_H_25_O_6_ |  |  | 533.2093 | 371.1371 |  | 227.4, 278.3 |  | Qiu *et al*. 2010;  Yi *et al*. 2009 |
| 16 | Hispidulin^*^ | 14.538 | 301.0711 | C_16_H_13_O_6_ |  |  | 181.1224 |  | 299.0463 |  |  | 272.7, 335.6 |  | Qiu *et al*. 2010;  Yi *et al*. 2009 |
| 17 | Jaceosidin ^#^ | 14.810 | 331.0822 | C_17_H_15_O_7_ |  |  |  |  | 329.0639 |  |  | 272.1, 346.9 |  | Yi *et al*. 2009 |
| 18 | Acacetin ^#^ | 16.202 | 285.0785 | C_16_H_13_O_5_ | 285.1202 |  |  |  | 283.0886 |  |  | 269.1, 326.4 |  | Yi *et al*. 2009 |

**^*^** Identified compound based on an authentic standard and reported literature values.

^#^ Putatively annotated compounds based on the accurate mass and literature values reported for authentic samples by other laboratories.

**References**

Clifford MN, Johnston KL, Knight S, Kuhnert N (2003) Hierarchical scheme for LC-MSn identification of chlorogenic acids. Journal of Agricultural and Food Chemistry 51: 2900-2911.

Fu C, Xu Y, Zhao D, Ma F (2006) A comparison between hairy root cultures and wild plants of *Saussurea involucrata* in phenylpropanoids production. Plant Cell Reports 24: 750-754.

Qiu J, Xue X, Chen F, Li C, Bolat N, et al. (2010) Quality evaluation of snow lotus (*Saussurea*): quantitative chemical analysis and antioxidant activity assessment. Plant Cell Reports 29: 1325-1337.

Hokkanen J, Mattila S, Jaakola L, Pirttilä AM, Tolonen A (2009) Identification of phenolic compounds from lingonberry (*Vaccinium* vitis-idaea L.), bilberry (*Vaccinium* *myrtillus* L.) and hybrid bilberry (*Vaccinium* x *intermedium* Ruthe L.) leaves. Journal of Agricultural and Food Chemistry 57: 9437-9447.

Li C, Du H, Wang L, Shu Q, Zheng Y, et al. (2009) Flavonoid composition and antioxidant activity of tree peony (*Paeonia sectio*n moutan) yellow flowers. Journal of Agricultural and Food Chemistry 57: 8496-503.

Lin L, Harnly J (2010). Identification of the phenolic components of chrysanthemum flower (Chrysanthemum morifolium Ramat). Food Chemistry 120: 319-326.

Saito N, Toki K, Honda T, Kawase K (1988) Cyanidin 3-malonylglucuronylglucoside in bellis and cyaniding 3- malonylglucoside in dendranthema. Phytochemistry 27:2963-2966

Yi T, Chen H, Zhao Z, Jiang Z, Cai S, et al. (2009) Identification and determination of the major constituents in the traditional Uighur medicinal plant Saussurea involucrata by LC-DAD-MS. Chromatographia 69: 537-542.

Yu Z, Fu C, Han Y, Li Y, Zhao D (2006) Salicylic acid enhances jaceosidin and syringin production in cell cultures of Saussurea medusa. Biotechnology Letters 28: 1027-1031.
